# Identification of genes involved in inbreeding depression of reproduction in Langshan chickens

**DOI:** 10.5713/ajas.20.0248

**Published:** 2020-10-14

**Authors:** Qian Xue, Guohui Li, Yuxia Cao, Jianmei Yin, Yunfen Zhu, Huiyong Zhang, Chenghao Zhou, Haiyu Shen, Xinhong Dou, Yijun Su, Kehua Wang, Jianmin Zou, Wei Han

**Affiliations:** 1 Poultry Institute, Chinese Academy of Agricultural Science, Yangzhou 225000, China; 2 Science and Technology Innovation Center, Poultry Institute of Jiangsu Province, Yangzhou 225000, China

**Keywords:** Conservation, Gallus gallus, Inbreeding Depression, Reproduction, Transcriptome

## Abstract

**Objective:**

Inbreeding depression of reproduction is a major concern in the conservation of native chicken genetic resources. Here, based on the successful development of strongly inbred (Sinb) and weakly inbred (Winb) Langshan chickens, we aimed to evaluate inbreeding effects on reproductive traits and identify candidate genes involved in inbreeding depression of reproduction in Langshan chickens.

**Methods:**

A two-sample t-test was performed to estimate the differences in phenotypic values of reproductive traits between Sinb and Winb chicken groups. Three healthy chickens with reproductive trait values around the group mean values were selected from each of the groups. Differences in ovarian and hypothalamus transcriptomes between the two groups of chickens were analyzed by RNA sequencing (RNA-Seq).

**Results:**

The Sinb chicken group showed an obvious inbreeding depression in reproduction, especially for traits of age at the first egg and egg number at 300 days (p<0.01). Furthermore, 68 and 618 differentially expressed genes (DEGs) were obtained in the hypothalamus and ovary between the two chicken groups, respectively. In the hypothalamus, DEGs were mainly enriched in the pathways related to vitamin metabolism, signal transduction and development of the reproductive system, such as the riboflavin metabolism, Wnt signaling pathway, extracellular matrix-receptor interaction and focal adhesion pathways, including stimulated by retinoic acid 6, serpin family F member 1, secreted frizzled related protein 2, Wnt family member 6, and frizzled class receptor 4 genes. In the ovary, DEGs were significantly enriched in pathways associated with basic metabolism, including amino acid metabolism, oxidative phosphorylation, and glycosaminoglycan degradation. A series of key DEGs involved in folate biosynthesis (gamma-glutamyl hydrolase, guanosine triphosphate cyclohydrolase 1), oocyte meiosis and ovarian function (cytoplasmic polyadenylation element binding protein 1, structural maintenance of chromosomes 1B, and speedy/RINGO cell cycle regulator family member A), spermatogenesis and male fertility (prostaglandin D2 synthase 21 kDa), Mov10 RISC complex RNA helicase like 1, and deuterosome assembly protein 1) were identified, and these may play important roles in inbreeding depression in reproduction.

**Conclusion:**

The results improve our understanding of the regulatory mechanisms underlying inbreeding depression in chicken reproduction and provide a theoretical basis for the conservation of species resources.

## INTRODUCTION

Inbreeding—mating between related individuals—often occurs in small, isolated, or captive populations. A negative effect of inbreeding is inbreeding depression, which has become a major concern in evolution, ecology, and conservation biology research [1]. Inbreeding depression refers to a reduction in fitness or fitness-related traits due to inbreeding, such as a reduction in juvenile survival rate and reproductive success. Especially, in small-scale populations, inbreeding depression in reproduction has significant implications on the ability of populations to evolve and adapt to environmental changes and, eventually, their long-term viability [2]. Thus, inbreeding depression in reproduction is a concern in the conservation of rare and endangered species.

According to classical population genetic studies, inbreed ing depression is caused mainly by the phenotypic expression of multiple recessive deleterious mutations and a decreased contribution of over-dominant loci [3]. However, these two hypotheses do not conclusively explain all cases of inbreeding depression. For example, inbreeding depression was found to change with environmental stress in several studies [4], and this was suggested to be associated with the epigenetic modification of DNA [5] and conditional expression of genes [6]. Overall, the mechanisms of inbreeding depression are complex and, therefore, further research should be conducted on different aspects of inbreeding depression at different levels.

Variations in gene expression are pervasively observed among cells, tissues, individuals, populations, and species [7]. Furthermore, most phenotypic differences are related to the regulation of gene expression even in the absence of genetic variation. Previous studies have shown that inbreeding causes changes in the expression of a large numbers of genes [8]. Several studies on *Drosophila melanogaster* suggested that some of the transcriptomic alterations in inbred individuals were due to inbreeding depression, whereas some have been reported as protective responses against inbreeding depression [8,9]. Despite these studies, a detailed regulatory mechanism of transcriptomic alterations in inbreeding depression is not well understood. To the best of our knowledge, most previous studies on inbreeding depression were performed in plants [5] and some animal models such as *Mus musculus* [10], *Drosophila melanogaster* [6,8] and *Argopecten irradians* [11]. So far, studies on genome-wide changes in gene expression in inbred individuals and the molecular mechanisms of inbreeding depression at the transcriptome level in poultry are limited. RNA sequencing (RNA-Seq) is an effective approach to study genome-wide gene expression, and it has been widely used to identify differentially expressed genes (DEGs) regulating various biological processes [12].

In the present study, we aimed to identify a set of candidate genes responsible for inbreeding depression of reproduction in Langshan chicken, a Chinese native chicken breed. We developed strongly and weakly inbred (Winb) chickens; a reduction in the performance of reproductive traits was observed in the strongly inbred (Sinb) individuals. To explore the regulatory mechanism of inbreeding depression in reproduction at the transcriptome level in chicken, we collected tissues from the gonadal axis, including the hypothalamus and ovary, for RNA-Seq.

## MATERIALS AND METHODS

### Ethics statement

All animal experiments were performed in accordance with the protocol of the Animal Use Committee of the Chinese Ministry of Agriculture and were approved by the Animal Care Committee of Chinese Academy of Agricultural Sciences, Beijing, China, under permit No. SYXK(Jing)2019-0046. All efforts were made to minimize animal suffering.

### Development of strongly and weakly inbred Langshan Chickens

**Figure 1:**
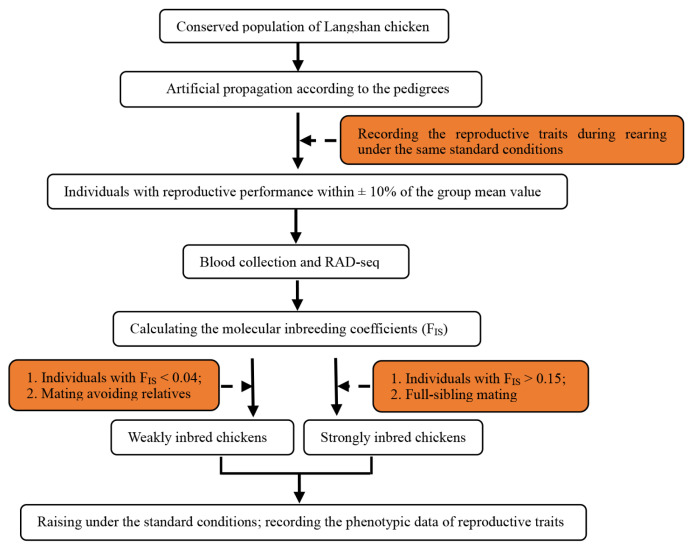
Flowchart of the development of strongly and weakly inbred chickens.

A Chinese native chicken breed, the Langshan chicken, conserved at the National Chicken Genetic Resources (Jiangsu, China), was selected as the study species. The conserved population of Langshan chicken was artificially propagated according to the pedigrees to obtain the F1 generation. We then selected 160 individuals from the F1 generation with similar reproductive performances (within±10% of the group mean value); they belonged to 10 families. Subsequently, restriction-site-associated DNA sequencing (RAD-Seq) was conducted on 43 of the 160 individuals (only one was sequenced to represent all the siblings). Based on the results of RAD-Seq, the molecular inbreeding coefficients (F_IS_) were calculated (ranging from−0.003 to 0.283). The F_IS_ of the full siblings was considered to be the same. Finally, individuals with F_IS_>0.15 (58 individuals from six families) were selected and crossed with full-sibling mates to obtain a Sinb F2 generation, and individuals with F_IS_<0.04 (12 individuals) were crossed, avoiding close relatives, to obtain a Winb F2 generation. The flowchart of generation of strongly and Winb chickens is presented in Figure 1.

### Individual selection and tissue collection

All the chickens in this study were raised under the same standard conditions in a closed chicken house with automatic ventilation, temperature control and humidity adjustment, and fed ad libitum with commercial complete feed. Phenotypic values for some of the reproductive traits (including age at the first egg, weight of the first egg, body weight at the first egg, and egg number at 300 days) were recorded. The IBM SPSS Statistics 20.0 software (International Business Machines Corporation, New York, USA) was used to analyze the data. A two-sample t-test was performed to estimate the differences between the two chicken groups for each trait. The traits with p<0.05 were considered to be significantly different.

Based on the estimated differences in reproductive traits between the two groups, three healthy chickens with reproductive trait values around the group mean values were selected from each of the groups. These six chickens were immediately humanely euthanized. The hypothalamus and ovary tissues were collected rapidly, snap-frozen in liquid nitrogen and then stored at −80°C until RNA extraction.

### RNA-seq library preparation and illumina sequencing

The total RNA from each sample was isolated using TRIzol regent (Invitrogen, Carlsbad, CA, USA). The purity, concentration, and integrity of the RNA were checked using a NanoDrop 2000 spectrophotometer (Thermo Fisher Scientific, Waltham, MA, USA) and an Agilent 2100 Bioanalyzer (Agilent Technologies, Santa Clara, CA, USA), respectively. The RNA integrity number of all samples was greater than 9.5. The polyA mRNA was recovered using oligo(dT) magnetic beads. Thereafter, the RNA was randomly fragmented and transcribed into cDNA. Sequencing adaptors were ligated to the fragments. Single-strand cDNA was then obtained using the Uracil-Specific Excision Reagent (USER) enzyme (NEB, Ipswich, UK). Polymerase chain reaction (PCR) amplification was performed to enrich the cDNA libraries with the following thermal profile: initial incubation at 95°C for 2 min, eight cycles of 95°C for 30 s, 65°C for 20 s, and 72°C for 45 s; and final incubation at 72°C for 7 min. Strand-specific RNA-Seq libraries were then generated. Sequencing was performed using the Illumina Hiseq 2500 instrument to generate 150-bp paired-end reads.

### Bioinformatic analysis of RNA-seq data

The raw data were subjected to quality control using FastQC (http://www.bioinformatics.babraham.ac.uk/projects/fastqc/). The reads that contained adapters, contaminations, low-quality bases, or undetermined bases were discarded. After passing the quality control step, the reads were mapped to the chicken reference genome (ftp://ftp.ncbi.nlm.nih.gov/genomes/all/GCF/000/002/315/GCF_000002315.5_GRCg6a/GCF000002315.5_GRCg6a_genomic.fna.gz) using HISAT2 [13]. The uniquely mapped reads by alignment were used for annotating transcripts and for determining gene expression levels using the fragments per kilobase per million reads (FPKM) method. The R psych package was used to perform the principle components analysis (PCA) based on the expression levels of all genes. The DESeq2 package was used to calculate differences in gene expression [14]. Genes with a false discovery rate (FDR) of <0.05 and fold change of ≥2 were considered to be DEGs between the two groups. The DEGs were then annotated using the gene ontology (GO) and the Kyoto encyclopedia of genes and genomes (KEGG) databases. Functional enrichment analysis was performed using the classic algorithm and Fisher’s exact test [15]. The GO terms and KEGG pathways with p<0.05 were considered significantly enriched.

### Validation of gene expression by real-time reverse transcription-quantitative polymerase chain reaction

Three DEGs were randomly selected from each of two tissues and were quantified by quantitative polymerase chain reaction (qPCR) to validate the RNA-Seq data. Primer pairs were designed using Primer Premier 5.0 software (PREMIER Biosoft, San Francisco, CA, USA) (Supplementary Table S1). The validation was performed with the same samples used for the RNA-Seq analysis. The total RNA was reverse-transcribed into cDNA using the PrimeScript RT Master Mix kit (TaKaRa Biotechnology Co Ltd, Dalian, China). qPCR was conducted on the Applied Biosystems 7500 real-time PCR system (Applied Biosystems, Foster City, CA, USA), and the reaction mixture with a total volume of 20 μL contained 10 μL of SYBR1 Premix Ex Taq (2×), 0.4 μL of ROX Reference Dye II (TaKaRa Biotechnology Co, Ltd., China), 0.4 μL of each primer (10 μM), 6.8 μL of RNase-free water, and 2 μL of cDNA.

The PCR conditions were as follows: initial denaturatio n at 95°C for 30 s, and 40 cycles of denaturation at 95°C for 5 s and annealing at 60°C for 34 s. Gene expression levels were normalized to that of β-actin to obtain relative expression levels using the 2^−ΔΔCt^ method [16].

### Graphic drawing

Several types of software were used for generating the figures, such as GraphPad Prism (GraphPad Software Inc., San Diego, CA, USA), Microsoft Office 2013 (Microsoft Corporation, Washington, USA), R package, and the cloud platform of Omicshare (https://www.omicshare.com/tools/).

## RESULTS

### Differences in reproductive traits between the Sinb and Winb chickens

As expected, significant differences existed in most of the reproductive traits between the Sinb and Winb chickens, especially for age at the first egg and egg number at 300 days (p<0.01). We also observed apparently reduced reproductive performance in Sinb chickens compared to Winb chickens (Table 1).

### Overview of RNA-seq data

A total of 12 samples from 4 groups were used for RNA-Seq. After quality control, 177.68 Gb clean data were finally obtained. An average of 54,301,961 and 41,131,453 clean reads were acquired for the ovaries of Winb and Sinb chickens (Winb_O, Sinb_O), respectively, while 54,775,767 and 47,209,256 clean reads were acquired for the hypothalamus tissues of the two groups (Winb_H, Sinb_H). The percentage of bases with quality value of ≥Q30 was no less than 89.26% for each sample. An average of 88.27% reads were mapped to the reference genome (Supplementary Table S2). A PCA analysis was performed to confirm the accuracy and reliability of the collected samples. The result showed that the replicates from the same groups clustered relatively closely (Supplementary Figure S1). Thus, the sample collections were reliable, and the sequencing data met the requirements for the subsequent analysis.

### Identification of differentially expressed genes

**Figure 2:**
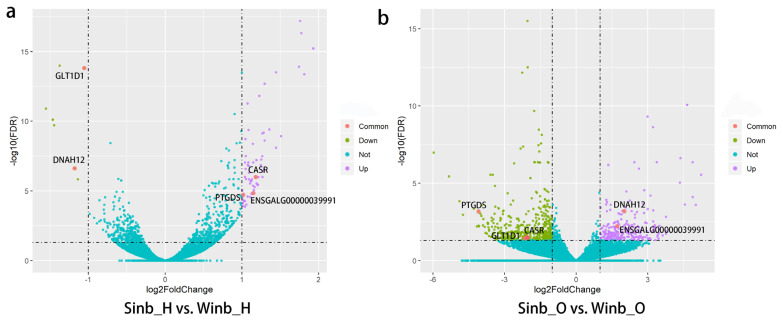
Volcanic map of the analysis of differentially expressed genes (DEGs). Blue dots indicate genes with no significant differences. Purple, green, and pink dots indicate upregulated, downregulated and the common DEGs in each comparison, respectively.

The expression levels of all genes were calculated as the FPKM using DESeq2. A comparison of gene expression in each of two tissues between the Sinb and Winb chickens was performed. We identified 68 DEGs between Sinb_H and Winb_H (Sinb_H vs Winb_H), and 618 DEGs between Sinb_O and Winb_O (Sinb_O vs Winb_O) (Supplementary Table S3-S4). Of the 68 DEGs in the hypothalamus, 61 genes were upregulated, and 7 genes were downregulated. Among the 618 DEGs in the ovary, we found 186 genes were upregulated and 432 genes downregulated (Figure 1). Five DEGs were commonly identified in both tissues, including calcium-sensing receptor, prostaglandin D2 synthase 21 kDa (PTGDS), ENSGALG000 00039991, glycosyltransferase 1 domain containing 1, and dynein, axonemal, heavy chain 12 (Figure 2).

DEGs with extremely significant differences in the com parisons are interesting as they may play important roles in related biological processes. As a result, the top five most differentially expressed genes in each of the two tissues ranked by FDR values were analyzed and obtained, for example, stimulated by retinoic acid 6 (*STRA6*), nephroblastoma overexpressed and deuterosome assembly protein 1 (*DEUP1*) genes in the hypothalamus and Mov10 RISC complex RNA helicase like 1 (*MOV10L1*), ribosomal protein S29, and ENSGALG00000002431 genes in the ovary (Table 2).

### Validation of differentially expressed genes

**Figure 3:**
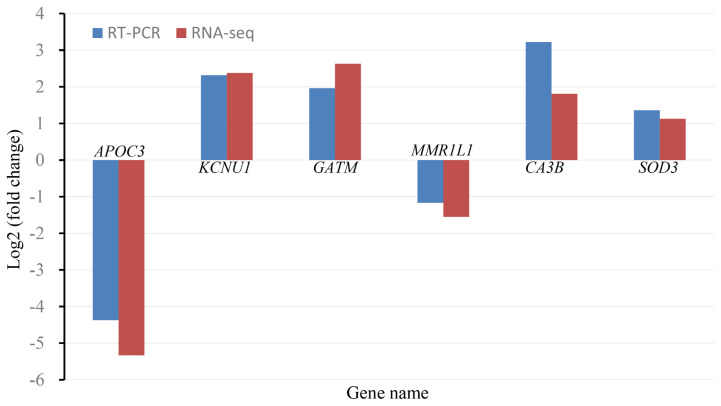
Quantitative polymerase chain reaction (qPCR) validation of differentially expressed genes (DEGs). Changes of expression levels for each gene between Sinb and Winb groups were calculated as log2(fold change). Real-time reverse transcription (RT)-qPCR results were consistent with the RNA-Seq results regarding the direction of changes in the expression level of DEGs.

To validate the accuracy of DEGs detected by RNA-Seq, we used real-time reverse transcription-qPCR (RT-qPCR) to evaluate the expression levels of six DEGs (apolipoprotein C3, potassium calcium-activated channel subfamily U member 1, glycine amidinotransferase from the ovary and macrophage mannose receptor 1-like, carbonic anhydrase 3B, superoxide dismutase 3 from the hypothalamus). It was found that the RT-qPCR results were consistent with the RNA-Seq results regarding the direction of changes in the expression level of DEGs (Figure 3), which confirmed the validity of the data from RNA-Seq.

### Functional analysis of differentially expressed genes

**Figure 4:**
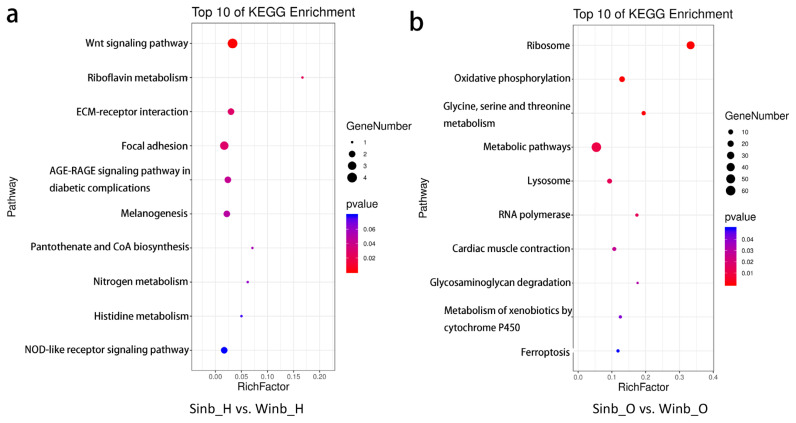
Kyoto encyclopedia of genes and genomes enrichment analysis of differentially expressed genes (DEGs) in the two comparisons. (a) The top 10 most enriched pathways in hypothalamus. (b) The top 10 most enriched pathways in ovary.

**Figure 5:**
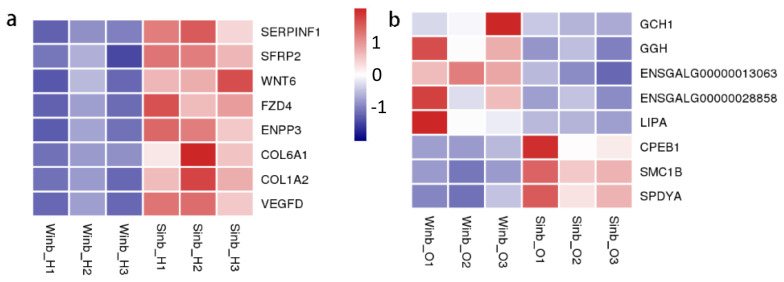
Heatmap of some of differentially expressed genes (DEGs) in hypothalamus (a) and ovary (b). The colors of bricks from navy to white to red represent the expression levels from low, medium to high.

The DEGs identified in the hypothalamus and ovary tissues between the Sinb and Winb chickens may be important for the regulation of inbreeding depression in chicken reproduction. Thus, to further explore the biological function of these genes, functional annotation and enrichment analysis were performed using the GO and KEGG databases. In the hypothalamus, five GO terms were significantly enriched (p< 0.05) by the DEGs, which were mainly related to the extracellular matrix (ECM) and extracellular region (Supplementary Figure S2). KEGG enrichment analysis of the DEGs revealed that 6 pathways were significantly enriched (p<0.05) (Figure 4a; Supplementary Table S5), including the Wnt signaling pathway, riboflavin metabolism, ECM-receptor interaction, focal adhesion, advanced glycation end-products and receptor for advanced glycation end-products signaling pathway in diabetic complications and melanogenesis. A total of eight DEGs were involved in these pathways, including serpin family F member 1 (SERPINF1), secreted frizzled related protein 2 (SFRP2), Wnt family member 6 (WNT6), frizzled class receptor 4 (FZD4), ectonucleotide pyrophosphatase/phosphodiesterase 3 (ENPP3), collagen type VI alpha 1 chain, collagen type I alpha 2 chain, and vascular endothelial growth factor D (Figure 5a).

In the ovary tissues, the DEGs were significantly enriched in GO terms mainly related to ribosomes and components of membranes, and a total of 29 GO terms were significantly enriched (p<0.05) (Supplementary Figure S3). The results of KEGG enrichment analysis showed that DEGs were significantly enriched in 10 pathways (p<0.05) (Figure 4b; Supplementary Table S6). These pathways were mainly related to the process of amino acid metabolism, oxidative phosphorylation, and glycosaminoglycan degradation. In addition, five key pathways related to sex hormone secretion and oocyte development were also enriched, namely, folate biosynthesis, steroid hormone biosynthesis, steroid biosynthesis, oocyte meiosis and progesterone-mediated oocyte maturation, including GTP cyclohydrolase 1 (*GCH1*), gamma-glutamyl hydrolase (*GGH*), ENSGALG00000013063, ENSGALG00000028858, lipase A, cytoplasmic polyadenylation element binding protein 1 (*CPEB1*), structural maintenance of chromosomes 1B (*SMC1B*), and speedy/RINGO cell cycle regulator family member A (*SPDYA*) genes (Figure 5b). These pathways and DEGs may play important roles in the regulation of inbreeding depression in reproduction in Langshan chickens.

## DISCUSSION

In the present study, by comparing the reproductive traits of Sinb and Winb chickens, we found a significant inbreeding depression of reproduction in the Sinb group, especially for the traits of age at the first egg and egg number at 300 days (p<0.01), which are the most representative in reflecting reproductive performance of female chickens. To explore the molecular mechanism underlying this phenomenon, transcriptomes of tissues from the gonadal axis, such as the hypothalamus and ovary, were compared between Sinb and Winb chicken groups. A total of 68 and 618 DEGs were identified in hypothalamus and ovary, respectively. Of these DEGs, five genes were found to co-exist in both tissues, which inferred that they play important regulatory roles in the gonadal axis. CARS, a calcium-sensing receptor, is central to extracellular calcium homeostasis, which is important in many physiological processes such as secretion, neurotransmission, and muscle contraction. A past study showed that CARS played a critical role in early embryogenesis [17]. In our study, CARS was differently expressed in both tissues, indicating it may affect the reproductive performance of Sinb Langshan chickens by regulating neurotransmission and endocrines of the gonad axis. PTGDS, which codes prostaglandin D synthase and catalyzes the conversion of prostaglandin H2 to prostaglandin D2, has been reported to be a specific gene involved in male chicken testicular development [18]. As a carrier for lipophilic molecules such as retinoids and thyroid hormones, altering PTGDS levels might influence vitamin A and thyroid hormone availability, and in turn have impacts on diverse biological processes. Thus, the differential expression of these genes in both tissues was inferred to regulate the reproduction of Sinb chicken by complicated biological processes, such as metabolism and secretion.

In addition to PTGDS, MOV10L1, and *DEUP1* were also reported to be specifically expressed in testicular tissue [19,20]. The expressions of MOV10L1 and *DEUP1* between the two chicken groups were extremely different, and they were in the top five ranked by FDR values. MOV10L1 participates in piwi-interacting RNA biogenesis and protects mouse male fertility, enhancing the proliferation capacities of spermatogonial progenitor cells [19]. A study on human tissue-specific expression reported that *DEUP1* was specifically highly expressed in testicular tissues, indicating *DEUP1* played an important role in male fertility [20]. Although our study was conducted on female chickens, as we know, a relatively large overlap exists between male and female fertility genes. So, it is speculated that these genes may enhance functions related to male fertility, which affect the reproduction of female chickens. However, the identification of these DEGs related to male fertility suggest that the reproduction of male chickens may be more sensitive to inbreeding effect, which points to a new direction for future research.

In the hypothalamus, several DEGs involved in vitamin metabolism were identified, such as STRA6 and ENPP3. STRA6, a cell-surface receptor for the retinol-binding protein, catalyzed vitamin A (retinol) influx, efflux, and exchange, which is involved in diverse biological processes [21]. Based the GO annotation, STRA6 is involved in the biological process of female genitalia development. In our study, STRA6 was found among the top five DEGs, suggesting it may play a critical role in the regulation of inbreeding depression of reproduction in Langshan chickens. Riboflavin, namely vitamin B_2_, has been shown to be essential for good hatchability of eggs. Furthermore, accurate riboflavin supplementation for rats was important for most of the reproductive cycle [22]. KEGG enrichment analysis of DEGs in hypothalamus showed that ENPP3 was significantly enriched in the pathway of riboflavin metabolism, which indicates a probable disorder of riboflavin metabolism in the Sinb chicken groups resulting in depressed reproduction.

Several DEGs in the hypothalamus were enriched in GO terms and KEGG pathways associated with ECM and extracellular region, such as ECM-receptor interaction and focal adhesion. Previous studies have shown that the brain’s ECM plays a crucial role in network formation, development, and regeneration of the central nervous system [23]. The interactions of neurons with ECM are important in directing the formation of precise neuronal networks during the development of the nervous system. In addition, focal adhesions have been considered as sites of transmembrane communication between the extracellular environment and the cytoplasm, or links between the ECM and the cytoskeleton. Therefore, it is inferred that high inbreeding in Langshan chickens would affect the formation and development of reproduction-related neuronal networks, leading to changes in signal transduction and neural regulation, and then influencing reproductive performance. Four DEGs (SERPINF1, SFRP2, WNT6, and FZD4) in the hypothalamus were found significantly enriched in the Wnt signaling pathway, which is a complex signaling pathway composed of Wnt, its receptors and regulators to regulate cell differentiation and participate in developmental processes. Studies in mammals have shown that Wnt signaling pathways are also involved in the development of the reproductive system such as the formation of the Mullerian duct and its derivatives, the development of ovarian follicules and mammary glands during pregnancy, ovulation and luteinization, and the establishment of a normal pregnancy [24]. Thus, it was inferred that these four DEGs may regulate the development of the reproductive system of Langshan chickens and affect the reproduction of Sinb chickens.

In the ovary, five key pathways related to sex hormone secretion and oocyte development were enriched, including folate biosynthesis, steroid hormone biosynthesis, steroid biosynthesis, oocyte meiosis and progesterone-mediated oocyte maturation, which may play critical roles in the regulation of inbreeding depression of reproduction in Langshan chickens. Hebert et al [25] studied laying hens and found that egg production was reduced in the absence of folate in the diet. GCH1 and GGH are the key enzymes in the folate pathway [26,27]. GCH1 converts guanosine triphosphate into pterin, which is subsequently incorporated into folate derivatives [26]. A previous study showed that GGH regulates the biosynthesis of folic acid in cells and promotes the hydrolysis and outflow of folate and folate antagonists from cells [27]. In our study, these two genes were both downregulated in Sinb chickens, suggesting they may reduce the egg production of Sinb chickens by affecting biosynthesis and derivation of folic acid. CPEB1, SMC1B, and SPDYA were found enriched in progesterone-mediated oocyte maturation and oocyte meiosis pathways, which were significantly highly expressed in the Sinb group. A recent study in a case-control analysis showed that significant enrichment of CPEB1 is possibly involved in primary ovarian insufficiency pathogenesis [28]. SMC1B is a meiosis-specific cohesin subunit that is essential for sister chromatid cohesion and DNA recombination. Previous studies have shown that SMC1B maintains the correct meiotic progression in mouse oocytes [29]. SPDYA (Spy1) is a new discovered cell cycle protein capable of promoting cell proliferation dependent on cyclin-dependent kinase-2 activation. A past study indicated Spy1 was probably associated with the proliferation of the epithelial ovarian cancer cells [30]. Therefore, these three genes may cause inbreeding depression of reproduction in Sinb chickens by affecting ovarian function or oocyte meiosis.

In addition, many DEGs identified in the ovary were found enriched in metabolism-related pathways, such as glycine, serine, and threonine metabolism, oxidative phosphorylation and glycosaminoglycan degradation. We speculate that high inbreeding in the population of Langshan chickens affects the basic metabolism, indirectly affects ovarian development, and leads to a decrease in reproductive performance. This finding provides a new direction to further explore the effects of inbreeding on the reproductive performance of poultry.

To our current knowledge, this is the first report on the identification of genes involved in inbreeding depression of chicken reproduction. The results of this study will improve our understanding of the regulatory mechanisms underlying inbreeding depression in chicken reproduction and provide a theoretical basis for the conservation of species resources. More efforts are needed to study the detailed molecular mechanisms of these genes in the regulation of inbreeding depression in chicken reproduction, which will be a challenge in the future.

## Figures and Tables

**Table 1:** Differences in reproductive traits between the two chicken groups1)

Traits	Groups (number of individuals)2)		p-value3)

	Sinb (73)	Winb (44)	
Age at the first egg (d)	173±15	150±8	0.001
Egg number at 300 days	84±28	98±15	0.01
Weight of the first egg (g)	36.1±3.4	37.1±3.5	0.814
Body weight at the first egg (g)	1,753±149	1,708±156	0.036

1) Phenotypic values of each trait were represented as means±standard errors.

2) Sinb is strongly inbred, Winb is weakly inbred

3) Comparisons between the two chicken groups based on a two-sample t-test. The traits with p<0.05 were considered to be significantly different.

**Table 2:** Top five most differentially expressed genes ranked by false discovery rate values in each tissue

Tissues	Top 10 DEGs	Description	log2(fold change)	FDR
Hypothalamus	STRA6	Stimulated by retinoic acid 6	1.76	6.60E-18
	NOV	Nephroblastoma overexpressed	1.77	4.79E-17
	ENSGALG00000041885	-	1.93	5.98E-16
	ENSGALG00000040576	-	1.93	5.98E-16
	DEUP1	Deuterosome assembly protein 1	−1.37	1.04E-14
Ovary	ENSGALG00000002431	-	−2.04	3.28E-16
	RPS29	Ribosomal protein S29	−2.03	3.13E-13
	ENSGALG00000030318	-	4.65	8.47E-11
	ENSGALG00000030044	-	4.65	8.47E-11
	MOV10L1	Mov10 RISC complex RNA helicase like 1	2.99	4.87E-10

DEGs, differentially expressed genes; FDR, false discovery rate.
