# Supplementary material for: Identification of genes involved in inbreeding depression of reproduction in Langshan chickens
Source: Anim Biosci. 2020 Oct 14;34(6):975–84. doi: 10.5713/ajas.20.0248 (PMC8100482; doi:10.5713/ajas.20.0248)
Supplement: Supplementary file 1 [file ajas-20-0248-suppl.pdf]

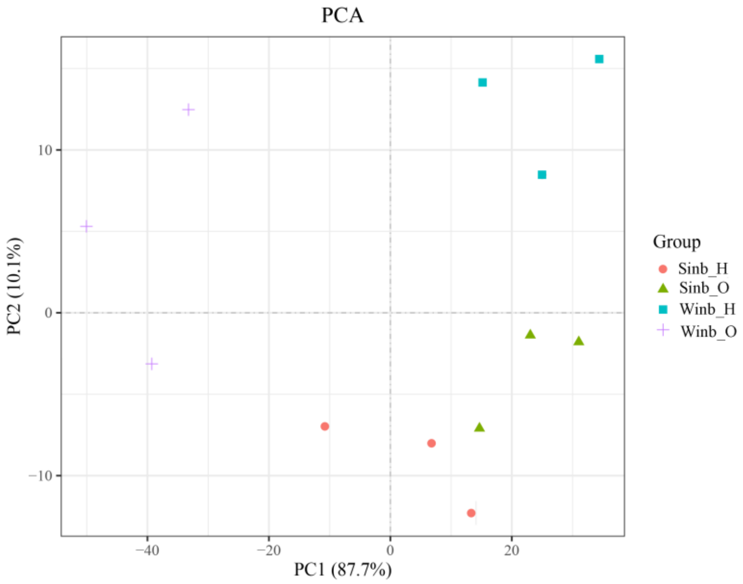

Figure S1. A principle components analysis (PCA)

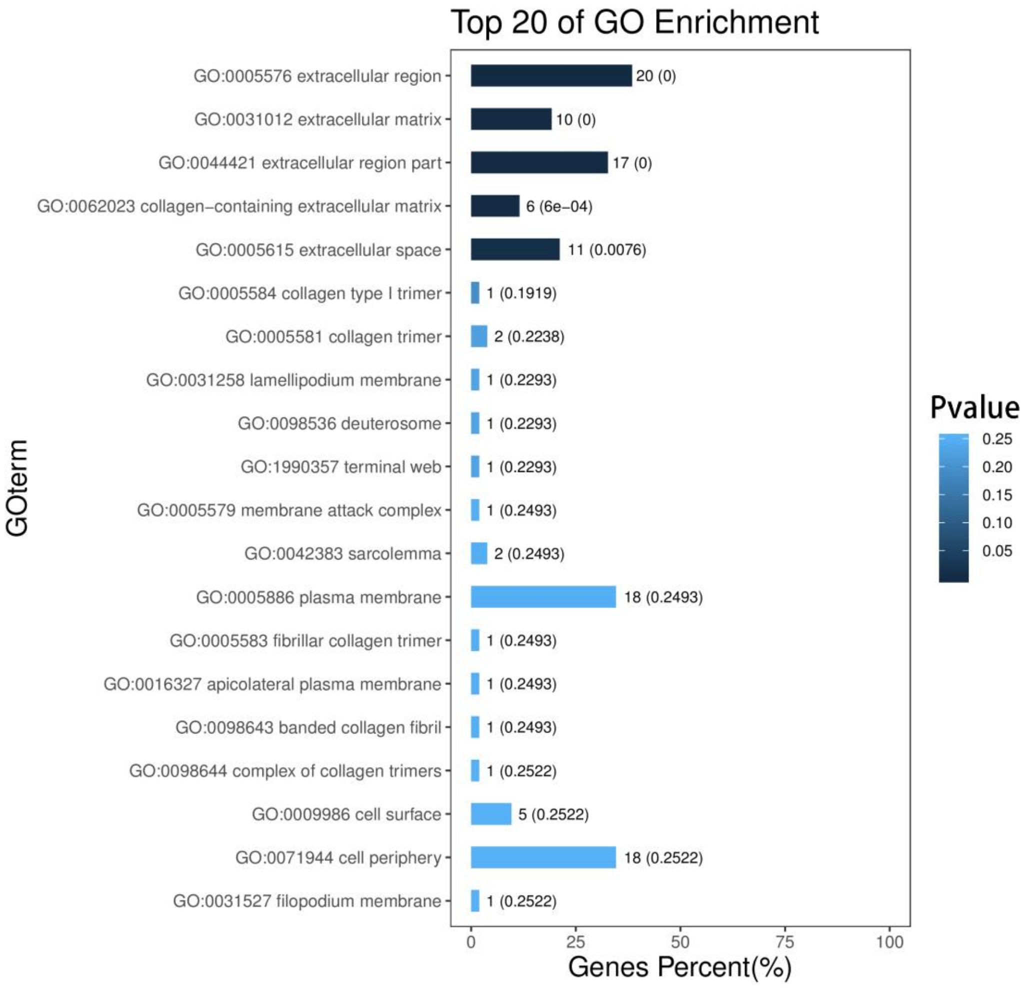

Figure S2. GO enrichment analysis of differentially expressed genes (DEGs) in hypothalamus

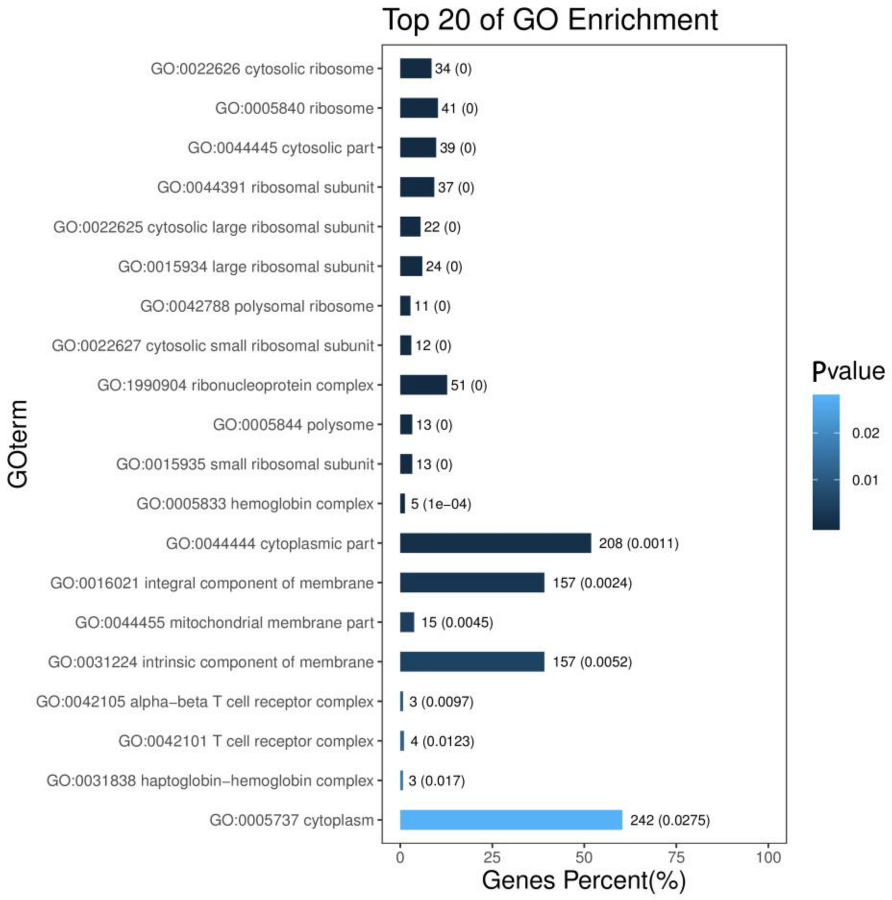

Figure S3. GO enrichment analysis of DEGs in ovary

Table S1 Primer pairs for qRT-PCR

Tissues	Gene	Primer	Sequence（5’ to 3’）	Annealing temp （℃）	Product size （bp）
Ovary	APOC3	P1	F:CCGAAGCTCCCGATAAGACA<br>R:TCTGACTCATGCACTGTGGT	58	106
	KCNU1	P2	F:TCACATCTTGGTCTGCGTCT<br>R:GGCCCAAGGAGAACAATGTC	58	127
	GATM	P3	F:GATGGGTGCAGCGATCTCT<br>R:AGCTCTTCCCACAATGACCT	59	158
Hypothalamus	MMR1L1	P4	F:ACTCCTTATTCTGGTCCTCG<br>R:GTCACTGCGTTCACTCCC	57	196
	CA3B	P5	F:AGAAGTGCACTATGACCGCT<br>R:TCAAACACAACTCTGCAGGT	58	105
	SOD3	P6	F:TGACAGAAACAGGAGCCGAT<br>R:CCAGTCACTTGTGGCTTGTC	59	184
Housekeeping genes	β-actin	P7	F: CAGCCATCTTTCTTGGGTAT<br>R: CTGTGATCTCCTTCTGCATCC	60	165

Table S2. Sequencing results and read alignment

Groups	Samples	Clean reads	Base number	%≥Q30	Reads aligned (%)
	Winb_01	57528672	17258601600	91.47	91.61
Winb_0	Winb_02	52212911	15663873300	89.26	85.47
	Winb_03	53164301	15949290300	91.64	89.9
	Sinb_01	37727863	11318358900	91.71	88.06
Sinb_0	Sinb_02	48872541	14661762300	91.94	89.03
	Sinb_03	36793957	11038187100	90.77	85.6
	Winb_H1	46974827	14092448100	92.82	91.61
Winb_H	Winb_H2	52212911	15663873300	89.26	85.47
	Winb_H3	65139563	19541868900	91.75	89.9
	Sinb_H1	43233881	12970164300	92.56	88.06
Sinb_H	Sinb_H2	45602090	13680627000	92.44	89.03
	Sinb_H3	52791799	15837539700	92.05	85.6

Table S3 The differentially expressed genes in the compasison of Sinb_H vs. Winb_H

#gene_id	gene_name	baseMeanA	baseMeanB	log2(Sinb_H/Winb_H)	FDR	regulated
ENSGALG00000009641	COL1A2	4483.704	15998.618	1.357771523	3.97E-10	up
ENSGALG00000028063	LOX	136.95417	458.2515	1.295392931	2.41E-09	up
ENSGALG00000003324	PRRX1	76.238655	261.05597	1.146775022	4.31E-06	up
ENSGALG00000014603	C1S	517.38819	1594.2109	1.265039539	7.55E-10	up
ENSGALG00000029015	TM6SF2	11.267143	78.229847	1.107572484	2.81E-05	up
ENSGALG00000002557	ADGRD1	1020.5734	2779.0297	1.228152644	1.55E-12	up
ENSGALG00000034868	KRT7	547.21403	2121.959	1.167274718	5.41E-06	up
ENSGALG00000021869	PODN	326.26173	903.87702	1.091351164	1.48E-06	up
ENSGALG00000029212	PADI2	94.352774	280.41496	1.108529132	2.89E-06	up
ENSGALG00000024379	--	20.975331	107.91885	1.144130265	1.71E-05	up
ENSGALG00000004695	ECM2	1176.7245	2743.4181	1.046986327	1.98E-09	up
ENSGALG00000026736	OGN	1027.3785	3648.1213	1.247107876	1.45E-07	up
ENSGALG00000037587	ENPP3	95.963905	430.68812	1.285891586	2.99E-07	up
ENSGALG00000034456	PRELP	436.88805	1273.4226	1.297461378	2.03E-13	up
ENSGALG00000009241	SFRP2	588.87904	1554.0477	1.007404055	2.64E-05	up
ENSGALG00000006758	C1QTNF5	106.57227	329.64421	1.016603623	0.000116	up
ENSGALG00000021525	--	701.0577	2219.4022	1.283715944	6.66E-10	up
ENSGALG00000026651	SCARA3	284.96811	842.31095	1.116178493	1.97E-06	up
ENSGALG00000003015	SERPINF1	1321.3343	3503.9381	1.144178442	4.21E-09	up
ENSGALG00000030886	PTGDS	18369.066	48481.163	1.014924524	1.94E-05	up
ENSGALG00000033171	TGM4	54.295201	187.06037	1.265263195	3.23E-08	up
ENSGALG00000041612	SLC7A13	509.15439	166.92491	-1.135809221	1.48E-06	down
ENSGALG00000041885	--	2.0857537	108.92961	1.931039284	5.98E-16	up
ENSGALG00000004893	TMEM119	101.96148	280.2	1.011978476	4.32E-05	up
ENSGALG00000038364	NOV	472.21105	2545.9303	1.773331492	4.79E-17	up
ENSGALG00000012298	SLC39A8	261.56405	795.17189	1.038690365	5.40E-05	up
ENSGALG00000040271	--	315.31141	74.20181	-1.44411993	1.99E-10	down
ENSGALG00000017242	FZD4	1268.1021	3060.8246	1.087723206	4.22E-10	up
ENSGALG00000019719	KRT19	683.19201	2570.2399	1.125491032	1.64E-05	up
ENSGALG00000012377	HNMT	203.50814	519.20926	1.084227531	9.04E-08	up
ENSGALG00000027423	SLC26A1	20451.603	54732.427	1.090803687	5.04E-07	up
ENSGALG00000005426	FREM1	547.84125	1270.3533	1.073348123	5.43E-12	up
ENSGALG00000009002	--	1399.3541	4203.951	1.189917437	6.23E-08	up
ENSGALG00000014835	C7	504.82625	1717.5553	1.252052436	5.70E-08	up
ENSGALG00000016473	OSR1	109.5987	676.71264	1.208744059	3.71E-06	up
ENSGALG00000012119	MARCO	183.13859	522.00141	1.009553399	7.45E-05	up
ENSGALG00000006175	DPEP1	15.598849	106.91464	1.443368449	7.98E-09	up
ENSGALG00000044263	DEUP1	4769.823	1529.6816	-1.373820928	1.04E-14	down
ENSGALG00000032262	--	1399.3541	4203.951	1.189917437	6.23E-08	up
ENSGALG00000030988	RBPMS2	53.162288	158.16018	1.029401101	5.62E-05	up
ENSGALG00000019509	CCDC102B	107.26716	322.1686	1.122551172	1.93E-06	up
ENSGALG00000007803	--	18.435465	152.16907	1.511550849	1.19E-09	up
ENSGALG00000016558	VEGFD	623.30646	1624.1005	1.090015105	1.66E-07	up
ENSGALG00000008006	CAPN6	593.47363	1507.8139	1.075440286	1.38E-07	up
ENSGALG00000004498	SLC2A10	151.02574	380.08517	1.004908465	9.38E-06	up
ENSGALG00000018557	SOD3	97.764905	294.43338	1.130266634	1.48E-06	up
ENSGALG00000038405	CASR	286.55116	948.88225	1.177818235	1.02E-06	up
ENSGALG00000011358	WNT6	78.638799	376.80522	1.040930596	0.000139	up
ENSGALG00000030603	ANTXR1	831.62702	2146.4306	1.053038198	1.44E-06	up
ENSGALG00000002679	GLT1D1	4849.3291	2172.105	-1.054920181	1.54E-14	down
ENSGALG00000015015	CYTL1	187.4148	760.15855	1.058690817	9.56E-05	up

ENSGALG00000004393	--	40.213357	271.15153	1.204673819	3.78E-06	up
ENSGALG00000027765	MMR1L1	866.96819	174.5874	-1.551358965	1.24E-11	down
ENSGALG00000039991	--	38.021806	170.75317	1.148297471	1.43E-05	up
ENSGALG00000002367	--	455.01696	1286.2138	1.149511797	9.04E-08	up
ENSGALG00000037975	CA3B	59.650547	728.10909	1.812128917	4.37E-14	up
ENSGALG00000004687	CENPP	202.89996	502.04815	1.049634067	2.66E-07	up
ENSGALG00000002201	PPL	1108.2777	2784.4442	1.041978486	1.02E-06	up
ENSGALG00000005601	--	1856.2137	600.80537	-1.178966686	2.40E-07	down
ENSGALG00000011274	DCN	12636.702	30447.151	1.04147184	9.31E-08	up
ENSGALG00000041346	CXCL12	2532.7591	6098.6349	1.052272357	3.17E-08	up
ENSGALG00000004778	SLC47A2	714.97394	4376.343	1.742564557	1.22E-14	up
ENSGALG00000043610	--	69.591313	261.48514	1.213514313	1.03E-06	up
ENSGALG00000040576	--	2.0857537	108.92961	1.931039284	5.98E-16	up
ENSGALG00000005974	COL6A1	275.18786	1221.3466	1.276467489	3.89E-07	up
ENSGALG00000010258	LTBP2	1152.9818	3993.3598	1.444401716	3.16E-14	up
ENSGALG00000001449	STRA6	149.53525	751.6444	1.760068326	6.60E-18	up
ENSGALG00000031441	--	254.12472	59.233054	-1.463721521	7.85E-11	down

Table S4 The differentially expressed genes in the compasison of Sinb_O vs. Winb_O

#gene_id	gene_name	baseMeanA	baseMeanB	log2(Sinb_O/Winb_O)	FDR	regulated
ENSGALG00000007863	COX7B	10428.06	2253.4038	-2.022775803	0.03097	down
ENSGALG00000005338	RPS21	29014.27	9390.5381	-1.605070473	4.39E-07	down
ENSGALG00000038882	--	231.9179	101.79644	-1.153257272	0.041401	down
ENSGALG00000003789	FAM78A	1996.053	594.27889	-1.65324182	0.029765	down
ENSGALG00000045441	C20orf27	2105.381	607.69482	-1.674802031	0.048836	down
ENSGALG00000028928	LCAT	3916.029	251.97768	-3.419294865	0.000956	down
ENSGALG00000013063	SRD5A1	241.2503	90.391841	-1.369291937	0.016486	down
ENSGALG00000015195	RPS15	96091.74	44046.472	-1.109485331	0.002442	down
ENSGALG00000042163	FAM136A	1007.414	334.38913	-1.573493108	3.45E-08	down
ENSGALG00000035825	--	3055.616	69.02038	-3.904637734	0.005373	down
ENSGALG00000019277	SLCO1B3	1011.57	12.320956	-4.081694457	0.005701	down
ENSGALG00000026005	DIO2	4514.969	321.79943	-3.080437044	0.01636	down
ENSGALG00000012005	LRRC9	57.58177	305.42727	2.19775357	0.01636	up
ENSGALG00000035309	--	6270.457	1032.1966	-2.422452029	0.00196	down
ENSGALG00000031214	MED9	1853.501	876.98862	-1.062344953	0.007033	down
ENSGALG00000013094	DDC	7229.95	116.72978	-3.520893106	0.029	down
ENSGALG00000033800	--	3019.06	1413.0247	-1.069486476	0.038588	down
ENSGALG00000038783	ANXA4	5486.566	2025.8444	-1.378396569	0.041532	down
ENSGALG00000006633	TMEM243	830.1423	259.81554	-1.642190624	4.13E-05	down
ENSGALG00000011484	CMTM8	193.9482	57.710524	-1.68280085	0.003655	down
ENSGALG00000028520	CST3	85369.47	14286.362	-2.318651205	0.019483	down
ENSGALG00000033376	APOH	24396.63	46.133148	-4.740471251	0.001112	down
ENSGALG00000005524	NTSR1	421.3964	97.538401	-1.973697489	0.014145	down
ENSGALG00000007047	GAL	1627.715	101.60472	-3.277823185	0.007216	down
ENSGALG00000033546	--	60.74341	16.901222	-1.706026472	0.048285	down
ENSGALG00000029480	TMEM138	2474.32	1183.8311	-1.038448398	0.045991	down
ENSGALG00000011290	RPL15	56516.12	18912.745	-1.563333573	3.50E-09	down
ENSGALG00000003966	RPL23A	59019.56	21094.721	-1.469790252	2.65E-08	down
ENSGALG00000014203	PARVG	2819.04	598.49209	-2.05471447	0.023328	down
ENSGALG00000031531	--	789.3204	3555.4305	2.035184731	0.008956	up
ENSGALG00000045747	--	4444.638	1648.3786	-1.377160324	0.031519	down
ENSGALG00000045065	--	31.63026	2.9203319	-2.933005319	0.007907	down
ENSGALG00000007875	--	500.9224	31.456144	-3.189551642	0.014181	down
ENSGALG00000002272	NOC4L	27470.2	81386.768	1.489171859	0.045456	up
ENSGALG00000013990	RPS12	65342.12	25841.836	-1.321917055	2.41E-05	down
ENSGALG00000030378	RPL11	61479.03	24803.567	-1.280772384	0.006034	down
ENSGALG00000027682	C12orf66	51375.66	21119.818	-1.259558645	0.002091	down
ENSGALG00000036558	--	6475.022	1074.0795	-2.322699109	0.021286	down
ENSGALG00000029444	CLK2	1079.278	154.68573	-2.636661779	0.000134	down
ENSGALG00000004297	DNAH1	1314.421	5516.2124	1.95823045	0.006428	up
ENSGALG00000027974	--	20.22481	116.7904	2.333797737	0.005145	up
ENSGALG00000014228	SMC1B	90.05612	808.36301	2.605683562	0.045226	up
ENSGALG00000035251	--	191.5451	70.645276	-1.395924277	0.00818	down
ENSGALG00000006919	POF1B	684.3263	2232.2988	1.649045787	0.00326	up
ENSGALG00000005978	RBP3	1062.26	4157.548	1.887454857	0.002116	up
ENSGALG00000034337	RHPN1	4991.487	1193.9681	-1.919903919	0.023131	down
ENSGALG00000034722	TLR2B	17450.17	4395.2039	-1.830346613	0.049384	down
ENSGALG00000016268	--	1939.722	6222.1011	1.620172315	0.006707	up
ENSGALG00000015372	ATP5I	4571.907	705.34178	-2.376024016	0.028413	down
ENSGALG00000023435	GATM	495.1474	3319.2876	2.632270882	1.14E-06	up
ENSGALG00000012123	PAX6	129.8045	8.8645914	-3.011265895	0.029	down

ENSGALG00000015419	PENK	3500.312	181.28974	-3.111568628	0.036804	down
ENSGALG00000014561	PTPN6	4924.433	1413.79	-1.683441312	0.046367	down
ENSGALG00000015179	CEP78	2103.136	5401.3621	1.347163303	6.69E-07	up
ENSGALG00000030031	TTPA	1982.09	328.79785	-2.38104895	0.006072	down
ENSGALG00000030853	--	249.548	579.96039	1.186211401	0.025275	up
ENSGALG00000010073	DDOST	24455.59	11227.274	-1.098562099	0.023951	down
ENSGALG00000008925	TMEM203	1054.096	488.27796	-1.089353512	0.013017	down
ENSGALG00000042997	--	177.464	50.856698	-1.694975966	0.03255	down
ENSGALG00000004875	PEMT	2583.41	981.63018	-1.344114376	0.03558	down
ENSGALG00000024031	CYB561	837.7366	49.929885	-3.041126499	0.036865	down
ENSGALG00000010208	CAPS2	437.7181	1242.0547	1.444465086	0.028438	up
ENSGALG00000018700	PLPP7	293.3314	61.099494	-2.098588644	0.012974	down
ENSGALG00000011565	TDRD9	689.1541	2845.8532	1.911127769	0.019159	up
ENSGALG00000041189	--	20.10566	240.35585	2.802176548	0.046402	up
ENSGALG00000038413	SURF6	4577.918	15675.762	1.695041031	0.012585	up
ENSGALG00000029571	TMEM55A	7320.447	18983.173	1.332013149	0.019895	up
ENSGALG00000003197	RPL7A	112088.9	54703.741	-1.016922565	0.018078	down
ENSGALG00000027950	TXNDC17	6818.212	2727.8633	-1.284838619	0.01703	down
ENSGALG00000004491	DMGDH	4223.981	1084.3178	-1.916117987	4.63E-06	down
ENSGALG00000012550	HMOX1	6037.375	1156.423	-2.143083173	0.035455	down
ENSGALG00000015617	RPS23	22465.54	10434.437	-1.078772802	0.043133	down
ENSGALG00000035854	--	61.42186	231.38389	1.843892156	0.001007	up
ENSGALG00000038884	SRL	238.6024	784.24663	1.631222582	0.025021	up
ENSGALG00000043603	CCL5	519.5175	40.635893	-3.180026416	0.002612	down
ENSGALG00000016589	SLC35F6	2436.198	1117.3473	-1.103515932	0.011892	down
ENSGALG00000016296	GFRAL	604.753	6.4622386	-3.741554273	0.018092	down
ENSGALG00000021143	RGS18	2169.449	389.41998	-2.219928029	0.030587	down
ENSGALG00000042633	--	6908.565	2208.4351	-1.561041061	0.036701	down
ENSGALG00000014570	LPCAT3	3633.329	1130.9158	-1.617966184	0.009483	down
ENSGALG00000028858	--	155.6156	46.020161	-1.650073654	0.040018	down
ENSGALG00000043481	--	8.314805	59.601175	2.484334958	0.019159	up
ENSGALG00000046169	PYM1	1990.408	734.09308	-1.384804258	0.029904	down
ENSGALG00000005345	CABLES2	29014.27	9390.5381	-1.605070473	4.39E-07	down
ENSGALG00000040772	--	656.8593	2753.0593	1.923134156	0.022589	up
ENSGALG00000045460	--	214.0581	611.62486	1.450223243	0.031519	up
ENSGALG00000038626	--	39.13577	271.87921	2.387820938	0.047042	up
ENSGALG00000014981	COX17	3725.244	1681.592	-1.115097569	0.049564	down
ENSGALG00000035111	PDRG1	3827.953	1242.9439	-1.551379815	0.022462	down
ENSGALG00000029544	--	1725.486	650.64208	-1.376642357	0.002151	down
ENSGALG00000008651	APMAP	13463.42	2821.7165	-2.055490423	0.03141	down
ENSGALG00000039296	--	26865.61	5777.4826	-2.004033693	0.047394	down
ENSGALG00000045394	--	15.66794	0	-3.369015244	0.045991	down
ENSGALG00000041054	FHDC1	1910.647	10125.638	2.237173322	0.005794	up
ENSGALG00000010502	SPATA6	16832.19	47524.084	1.430103797	0.043709	up
ENSGALG00000035445	HIST1H46L2	24968.72	9346.3501	-1.36955581	0.022589	down
ENSGALG00000032873	--	24.64718	156.52728	2.469481753	0.001679	up
ENSGALG00000042989	--	12763.73	2338.9329	-2.276201145	0.004816	down
ENSGALG00000023897	RGS21	0.509685	11.474942	3.049498087	0.04402	up
ENSGALG00000016128	B3GALT5	2451.464	281.96718	-2.704683356	0.014766	down
ENSGALG00000008623	TOR4A	2399.251	944.00524	-1.315103923	0.005638	down
ENSGALG00000029246	--	26865.61	5777.4826	-2.004033693	0.047394	down
ENSGALG00000006681	BRSK2	25313.06	2026.2013	-2.831137546	0.045728	down
ENSGALG00000042515	--	1939.722	6222.1011	1.620172315	0.006707	up
ENSGALG00000017090	POMP	13235.83	4084.6262	-1.615924151	0.02147	down

ENSGALG00000030016	CTSB	111564.1	38664.862	-1.500169859	0.000158	down
ENSGALG00000036355	C7orf73	2526.028	843.40275	-1.512438687	0.028137	down
ENSGALG00000013173	PYROXD1	7405.422	37448.045	2.148490297	0.016415	up
ENSGALG00000037241	KCNAB2	9962.638	633.17176	-3.129679714	0.019395	down
ENSGALG00000030030	RSPO2	433.8977	45.823617	-2.630647309	0.049209	down
ENSGALG00000010858	LRP2	2445.2	7009.4584	1.445870234	0.049947	up
ENSGALG00000000474	RPL36	48567.92	14132.845	-1.75173816	4.39E-07	down
ENSGALG00000005506	HESX1	2241.358	6052.9378	1.389026155	0.014104	up
ENSGALG00000035354	--	4.080562	38.352338	2.707712843	0.025319	up
ENSGALG00000036801	--	55.74455	8.6150318	-2.413363867	0.012974	down
ENSGALG00000000168	ADORA1	283.885	13.157579	-3.428108847	0.010048	down
ENSGALG00000038950	--	213.0228	6400.9393	4.372256832	2.38E-07	up
ENSGALG00000040182	GAMT	4444.638	1648.3786	-1.377160324	0.031519	down
ENSGALG00000032087	MC5R	4980.543	1358.2458	-1.775364721	0.015608	down
ENSGALG00000038479	--	47.31653	8.9077656	-2.172299723	0.031519	down
ENSGALG00000002397	PSPH	6973.505	777.07911	-2.623469727	0.040078	down
ENSGALG00000015192	--	4444.638	1648.3786	-1.377160324	0.031519	down
ENSGALG00000004438	ARSB	4223.981	1084.3178	-1.916117987	4.63E-06	down
ENSGALG00000010961	IGF2BP3	1864.945	6647.9837	1.724053277	0.030779	up
ENSGALG00000026364	ASAH1	23750	7684.4934	-1.5571845	0.020832	down
ENSGALG00000036097	CDH18	9029.505	326.3041	-3.443197157	0.018022	down
ENSGALG00000015616	ACOT12	170.2163	513.34186	1.528701609	0.016101	up
ENSGALG00000010526	OSTC	77926.63	26647.929	-1.528517879	4.69E-07	down
ENSGALG00000001618	OGFOD3	19038.56	57430.558	1.516154197	0.036804	up
ENSGALG00000000302	TNNT2	76.01153	525.78135	2.414116457	0.035773	up
ENSGALG00000044814	CCDC14	7323.52	28870.619	1.83197234	0.040078	up
ENSGALG00000042705	KCNK15	248.8265	23.586738	-2.809830889	0.024863	down
ENSGALG00000040907	--	3019.06	1413.0247	-1.069486476	0.038588	down
ENSGALG00000004671	ANKRD27	7735.397	18170.958	1.20175673	0.018166	up
ENSGALG00000027963	COX7C	16106.31	3133.0367	-2.154742074	0.02133	down
ENSGALG00000024485	BET1	2619.459	1240.4363	-1.060533067	0.01012	down
ENSGALG00000039224	HHLA1	79.35874	2.5502849	-3.668969985	0.007087	down
ENSGALG00000036093	PTPN7	1140.767	110.71332	-3.039158392	0.000597	down
ENSGALG00000009730	SLC38A4	10874.06	331.65723	-3.650435218	0.010048	down
ENSGALG00000007048	SYNM	16387.72	53469.053	1.639458254	0.00826	up
ENSGALG00000006891	ITIH5	7904.22	3639.9856	-1.089263391	0.047394	down
ENSGALG00000016565	CHRNA2	736.7485	23.038913	-3.888692929	0.002107	down
ENSGALG00000040263	--	13598.4	5347.8291	-1.325629358	0.00028	down
ENSGALG00000011295	SOCS2	368.0417	148.14545	-1.283499763	0.004772	down
ENSGALG00000038292	TMEM258	4359.829	1661.1431	-1.344170571	0.027773	down
ENSGALG00000027260	MC3R	57.46735	500.04855	2.727876727	0.010638	up
ENSGALG00000023953	--	11878.45	2673.3956	-2.019867118	0.008632	down
ENSGALG00000016232	RPL8	109421.2	46806.638	-1.209263155	0.000308	down
ENSGALG00000045205	--	10.50027	76.653615	2.588355181	0.006195	up
ENSGALG00000013098	MGST1	17642.8	3612.64	-2.136260424	0.007101	down
ENSGALG00000014508	CD38	4320.151	237.91888	-3.382011083	0.006541	down
ENSGALG00000001869	LINGO2	182.0646	11.313249	-3.063058937	0.029904	down
ENSGALG00000016477	--	6720.177	3201.4365	-1.056400558	0.001951	down
ENSGALG00000017068	KL	266.2254	29.246943	-2.818914771	0.005316	down
ENSGALG00000037464	WDR27	1521.16	5211.9989	1.678196349	0.028658	up
ENSGALG00000001416	ADRA1B	370.1777	73.588404	-2.106414135	0.034433	down
ENSGALG00000028451	MT4L	19051.43	2290.2072	-2.782275023	0.001425	down
ENSGALG00000026374	PFDN2	4981.358	1976.3515	-1.294100432	0.021151	down
ENSGALG00000005259	VIPR1	869.1166	286.96034	-1.56297914	0.000368	down

ENSGALG00000044709	--	72.33485	4.97579	-3.387433693	0.000334	down
ENSGALG00000044299	--	194.2243	713.6164	1.800243639	0.004596	up
ENSGALG00000012229	RPS29	26531.12	6398.4198	-2.027111498	3.13E-13	down
ENSGALG00000030397	TRIM27.1	125.2565	44.878656	-1.437295574	0.009653	down
ENSGALG00000037727	OSGEPL1	2100.746	814.19581	-1.318884739	0.034882	down
ENSGALG00000030420	--	500.9224	31.456144	-3.189551642	0.014181	down
ENSGALG00000000549	URAH	310.74	49.345129	-2.407083305	0.009515	down
ENSGALG00000043379	--	82286.98	35559.114	-1.195644685	0.000229	down
ENSGALG00000039197	--	821.2566	306.04154	-1.365345544	0.04309	down
ENSGALG00000042919	--	0.276083	41.859733	4.884509093	7.89E-05	up
ENSGALG00000031659	--	10476.83	953.5199	-2.857160897	0.022462	down
ENSGALG00000023411	CD180	213.9342	26.944605	-2.566735196	0.02738	down
ENSGALG00000020260	PGRMC1	67960.17	30854.765	-1.117424569	0.012204	down
ENSGALG00000046201	--	252.9333	1737.0909	2.51504321	0.007101	up
ENSGALG00000010218	--	549.6705	1153.9484	1.048952872	0.020261	up
ENSGALG00000032641	--	5973.079	976.7952	-2.354360394	0.016063	down
ENSGALG00000001117	PFKFB2	11878.45	2673.3956	-2.019867118	0.008632	down
ENSGALG00000044167	--	4.194841	51.361272	3.380756608	4.46E-07	up
ENSGALG00000009081	ZNF106	8733.405	22933.275	1.353469394	0.012135	up
ENSGALG00000013135	GALNT1	20324.51	9727.3888	-1.040104855	0.034285	down
ENSGALG00000004266	TNFAIP8L1	1323.585	473.90383	-1.432914097	0.013412	down
ENSGALG00000009803	MGST2	931.4131	92.486537	-2.928117161	0.004302	down
ENSGALG00000010357	P2RY1	5914.156	489.06165	-2.945831136	0.019606	down
ENSGALG00000030432	RASSF2	8074.49	1820.4878	-1.96737209	0.036865	down
ENSGALG00000016775	RPL31	48346.53	20305.29	-1.237897885	3.02E-05	down
ENSGALG00000036876	TMEM230	5241.415	2112.6374	-1.26817211	0.034602	down
ENSGALG00000035239	GLCCI1	7383.342	3344.6658	-1.125458824	0.002612	down
ENSGALG00000043654	--	4984.762	649.7062	-2.589036404	0.014487	down
ENSGALG00000037712	--	789.026	159.32539	-2.110304015	0.022462	down
ENSGALG00000037805	RPS19	31639.67	14801.988	-1.071393367	0.031519	down
ENSGALG00000003929	SYS1	1439.334	669.61146	-1.082605268	0.01636	down
ENSGALG00000013043	FAM173B	3550.372	9820.6675	1.419545986	0.01504	up
ENSGALG00000030920	APOC3	2035.845	15.719223	-5.327913104	3.61E-06	down
ENSGALG00000029617	COL17A1	886.7968	5439.3274	2.360413394	0.015256	up
ENSGALG00000043995	--	28115.68	8426.7755	-1.686843209	0.001047	down
ENSGALG00000030248	--	54.31527	281.11264	2.141911662	0.029044	up
ENSGALG00000038270	--	6086.216	18114.747	1.513096103	0.016415	up
ENSGALG00000013007	--	60.09013	343.45867	2.438893421	4.39E-07	up
ENSGALG00000004769	PSAP	836698.4	183281.11	-2.038622361	0.014181	down
ENSGALG00000039764	--	11014.05	667.13214	-3.196570388	0.015897	down
ENSGALG00000035691	--	63.63468	7.8176466	-2.639135284	0.016518	down
ENSGALG00000004924	OPNP	841.5316	40.414584	-3.172581672	0.032688	down
ENSGALG00000016222	NDP	1591.461	441.80969	-1.766256097	0.007537	down
ENSGALG00000016724	RGN	2653.301	474.56706	-2.229481108	0.02798	down
ENSGALG00000027176	--	81.33176	570.39652	2.436401149	0.032443	up
ENSGALG00000012748	ELOVL2	2552.809	13.601019	-3.651734003	0.026084	down
ENSGALG00000010090	FAM160A1	3493.096	14893.389	1.933229563	0.029904	up
ENSGALG00000015826	HTR1E	83.41917	12.707088	-2.332418115	0.047803	down
ENSGALG00000009026	--	1190.821	3858.3983	1.602542789	0.038554	up
ENSGALG00000040583	--	935.2843	323.2458	-1.514764628	4.58E-07	down
ENSGALG00000008127	SPI1	4822.883	970.63608	-2.08063669	0.042731	down
ENSGALG00000044328	--	127.4874	14.767645	-2.700617637	0.013661	down
ENSGALG00000014210	--	12246.96	31406.625	1.309688882	0.038588	up
ENSGALG00000010813	GOLGA5	33990.02	3300.8638	-2.698961337	0.047042	down

ENSGALG00000031341	--	367.6608	1306.0456	1.714449257	0.035455	up
ENSGALG00000040287	--	5.384138	57.521725	2.765410964	0.036272	up
ENSGALG00000002564	RGS4	9111.442	276.60848	-3.96641823	0.001301	down
ENSGALG00000028273	HBBA	5868.403	933.74121	-2.420486492	0.007104	down
ENSGALG00000030886	PTGDS	12691.68	354.18428	-4.092352634	0.00066	down
ENSGALG00000040878	TMEM208	3693.315	1115.2611	-1.629902688	0.036995	down
ENSGALG00000011093	AK7	1454.788	7110.6792	2.12587575	0.010672	up
ENSGALG00000007139	FAM107A	470.9567	191.44819	-1.266378374	0.012135	down
ENSGALG00000029862	--	350.6136	1871.9029	2.227552539	0.010048	up
ENSGALG00000036395	--	2524.698	296.59062	-2.576451265	0.041859	down
ENSGALG00000008447	SH2D1A	2356.522	297.37467	-2.723976841	0.001717	down
ENSGALG00000038141	NCF4	1336.055	300.34352	-2.012279638	0.012491	down
ENSGALG00000010811	LGMN	26789.79	8834.1018	-1.556775477	0.001951	down
ENSGALG00000037342	--	653.1772	1676.4364	1.328435608	0.004767	up
ENSGALG00000010187	SLC1A1	1681.814	116.51833	-3.148866487	0.011618	down
ENSGALG00000016321	ICK	1687289	350139.31	-2.216035062	4.33E-08	down
ENSGALG00000042066	--	64.04927	299.28356	2.096304435	0.004047	up
ENSGALG00000030356	OTOP1	35.08306	0.7400939	-3.293258521	0.043133	down
ENSGALG00000032002	RPL34	77926.63	26647.929	-1.528517879	4.69E-07	down
ENSGALG00000031786	BLVRA	12796.81	1481.4248	-2.824366355	0.001351	down
ENSGALG00000040882	HSPA4	18594.66	38382.137	1.035377215	0.000388	up
ENSGALG00000011203	HPSE	513.9143	83.917437	-2.272927419	0.049028	down
ENSGALG00000015410	BVES	419.1929	1208.2618	1.467842868	0.021703	up
ENSGALG00000017347	HBBR	5868.403	933.74121	-2.420486492	0.007104	down
ENSGALG00000035787	GABRR3	3.430313	32.426659	2.894541495	0.003544	up
ENSGALG00000011481	GPD1L	7025.295	2840.8459	-1.27316479	0.01284	down
ENSGALG00000037863	SEC61G	7882.841	2638.1448	-1.516456232	0.018133	down
ENSGALG00000042642	RARRES2	13065.82	6393.7029	-1.010648296	0.03141	down
ENSGALG00000045878	--	12.59354	210.55951	3.303163929	0.007101	up
ENSGALG00000004947	POLR3A	22933.77	56697.17	1.276998212	0.006378	up
ENSGALG00000029462	--	2404.662	5150.4034	1.081102808	0.007809	up
ENSGALG00000036327	NGEF	2728.655	229.83789	-2.841750381	0.036149	down
ENSGALG00000011994	SYNPO2	15655	41303.958	1.35174626	0.026009	up
ENSGALG00000033664	MOV10L1	301.0804	2582.437	2.992192403	4.87E-10	up
ENSGALG00000011418	CCR6	172.509	9.6639449	-3.470410677	0.002151	down
ENSGALG00000040827	USF1	511.21	1263.5787	1.286572671	0.000169	up
ENSGALG00000025755	SPTSSA	3369.394	1352.2219	-1.271496365	0.041532	down
ENSGALG00000044173	--	6.073788	94.727012	3.171527968	0.013133	up
ENSGALG00000043633	--	71.4144	31.590392	-1.145542772	0.038479	down
ENSGALG00000026311	C12orf75	1867.594	506.4888	-1.808045618	0.002739	down
ENSGALG00000026781	ALOX5AP	1219.795	98.787949	-3.171828644	0.001729	down
ENSGALG00000043428	FBXW4	5472.281	2582.0811	-1.059119015	0.034946	down
ENSGALG00000044241	--	17.64095	186.22041	3.116529689	6.37E-05	up
ENSGALG00000003445	MGST3	7307.737	2281.3123	-1.591317884	0.034946	down
ENSGALG00000045668	KCNU1	60.93422	396.62172	2.378330073	0.028009	up
ENSGALG00000011692	CDCA4	9679.583	4721.0021	-1.015733685	0.027862	down
ENSGALG00000039340	--	121.3628	13.993412	-2.677576452	0.020328	down
ENSGALG00000006300	LDHA	17571.65	4213.1811	-1.928055652	0.016421	down
ENSGALG00000013747	TAGAP	2088.105	443.02265	-2.072082083	0.015476	down
ENSGALG00000023571	TRIM59	5725.757	2030.417	-1.463933752	0.000703	down
ENSGALG00000020479	--	1476.269	478.56801	-1.542192742	0.039106	down
ENSGALG00000028363	SPG7	133582	58450.696	-1.183628131	4.39E-07	down
ENSGALG00000042232	PSMG4	2182.72	973.81859	-1.147840823	0.001616	down
ENSGALG00000006989	--	3209.887	9311.95	1.472510744	0.027867	up

ENSGALG00000016761	LYG2	407.2639	48.807128	-2.588666842	0.032555	down
ENSGALG00000033181	--	1959.423	680.17718	-1.478627639	0.008105	down
ENSGALG00000036743	MGAT3	35714.81	14374.542	-1.266737115	0.044941	down
ENSGALG00000013218	C3AR1	1051.648	95.866099	-2.994315973	0.005241	down
ENSGALG00000044761	--	3.185556	82.990361	3.761162689	0.001522	up
ENSGALG00000023025	--	2673.581	12994.312	2.114130802	0.012802	up
ENSGALG00000003589	VTN	15909.92	598.05741	-3.956649323	0.000273	down
ENSGALG00000006378	LIPA	13602.78	5358.1985	-1.301883234	0.025319	down
ENSGALG00000033943	HYI	858.1133	380.42136	-1.15125107	0.007907	down
ENSGALG00000015339	RPL24	35372.63	14240.608	-1.292756918	0.000368	down
ENSGALG00000032534	OBSCN	1189.71	3749.5912	1.602941087	0.003612	up
ENSGALG00000044739	--	22.90273	226.40232	2.996120318	0.000472	up
ENSGALG00000013828	GALM	2393.563	1156.4999	-1.025140805	0.047816	down
ENSGALG00000026214	LRTM2	2194.861	80.978974	-3.699989291	0.004279	down
ENSGALG00000002645	PIWIL1	1673.923	5400.3496	1.62698808	0.006957	up
ENSGALG00000009301	CHRNA1	205.119	636.91632	1.580519479	0.003883	up
ENSGALG00000005903	NRIP3	6092.957	158.26923	-3.494127626	0.023069	down
ENSGALG00000031956	--	70.031	14.443903	-2.059720457	0.045885	down
ENSGALG00000027789	RAB37	609.8748	49.115158	-3.242531819	0.000431	down
ENSGALG00000008620	RPL39	36676.73	17831.588	-1.02885657	0.001275	down
ENSGALG00000013342	GBX2	513.1407	56.299064	-2.645949981	0.036804	down
ENSGALG00000031375	CHST7	6410.14	3036.4889	-1.051715034	0.046134	down
ENSGALG00000001607	C17orf62	4626.003	2006.4087	-1.188515403	0.00066	down
ENSGALG00000002336	--	27470.2	81386.768	1.489171859	0.045456	up
ENSGALG00000036942	KCNA3	618.1977	40.407161	-3.29914012	0.004173	down
ENSGALG00000033907	--	156.1939	606.36694	1.819362421	0.033739	up
ENSGALG00000030318	--	39.92569	1355.9274	4.652509807	8.47E-11	up
ENSGALG00000041634	ACTG2	1441.16	31490.185	3.122864418	0.042926	up
ENSGALG00000042523	--	57.75308	10.859628	-2.239011447	0.005227	down
ENSGALG00000014673	RFESD	99.8781	21.902489	-2.057736722	0.005321	down
ENSGALG00000029897	RPL30	62996.33	30877.539	-1.016817331	0.002087	down
ENSGALG00000002157	RPS17	104105.6	47195.063	-1.111164854	0.04284	down
ENSGALG00000041662	OPN4-1	157.1224	412.026	1.356208252	0.004337	up
ENSGALG00000005519	TLL2	3096.551	11038.011	1.755388409	0.006469	up
ENSGALG00000012291	POLR2F	7510.591	2828.5446	-1.371913721	0.007101	down
ENSGALG00000005815	TMEM41B	10692.87	4924.9456	-1.096022431	0.016997	down
ENSGALG00000011459	--	172.1696	472.30719	1.427261718	0.000531	up
ENSGALG00000028031	C1QL1	501.3197	22.132477	-3.528255998	0.0065	down
ENSGALG00000020002	SLC22A3	3138.232	11794.098	1.783246985	0.033896	up
ENSGALG00000043234	HBA1	5998.585	1057.125	-2.31870518	0.005185	down
ENSGALG00000008317	SDSL	155.4586	17.826518	-2.660462376	0.022592	down
ENSGALG00000014722	KLRD1	28.36375	0.7400939	-4.006395767	0.000957	down
ENSGALG00000040557	TFEC	362.1793	97.826842	-1.768461535	0.031519	down
ENSGALG00000005094	GRAP	394.0757	63.189803	-2.343269214	0.025904	down
ENSGALG00000014878	MAN1A1	15501.28	5877.4052	-1.365838677	0.004494	down
ENSGALG00000016554	ACE2	389.2672	13.595114	-3.320849054	0.030385	down
ENSGALG00000009528	TMEM192	1346.302	3551.356	1.352790584	0.022479	up
ENSGALG00000001330	ATP5MC1	23433.04	4556.4875	-2.117772944	0.041512	down
ENSGALG00000037690	--	93244.47	19440.079	-2.07130408	0.02546	down
ENSGALG00000046427	--	204.4546	13.681463	-3.059591125	0.02403	down
ENSGALG00000038544	--	6720.177	3201.4365	-1.056400558	0.001951	down
ENSGALG00000041038	--	2.357306	51.380659	3.48166856	0.005794	up
ENSGALG00000030824	SF3B5	3128.853	1391.8476	-1.143991182	0.015066	down
ENSGALG00000006841	POLR2L	1722.763	681.85395	-1.286631058	0.048285	down

ENSGALG00000004630	--	487.9909	2320.8697	2.125388642	0.002793	up
ENSGALG00000045563	--	279.6232	1720.393	2.33070204	0.025829	up
ENSGALG00000010116	DNAH8	240.2791	1003.933	1.899483348	0.037089	up
ENSGALG00000009511	--	171.6251	1412.4693	2.85365314	2.85E-05	up
ENSGALG00000017040	--	28035.21	608.03061	-4.290358907	0.000448	down
ENSGALG00000036403	--	28.04827	0	-4.157976845	0.006309	down
ENSGALG00000000868	RNF121	4545.306	2173.5785	-1.049596096	0.004047	down
ENSGALG00000009596	TMEM117	6790.23	489.69429	-3.069634254	0.016497	down
ENSGALG00000013709	TIMM21	1597.491	711.5016	-1.137757344	0.032437	down
ENSGALG00000001942	SPDL1	4033.773	10776.377	1.359545193	0.045226	up
ENSGALG00000007611	RPL35A	28115.68	8426.7755	-1.686843209	0.001047	down
ENSGALG00000013546	--	228.1606	43.280382	-2.144091701	0.039605	down
ENSGALG00000016653	FAM167A	1240.153	183.173	-2.455733253	0.016497	down
ENSGALG00000039885	--	385.8341	1402.025	1.73422734	0.043861	up
ENSGALG00000006911	TMC5	564.9332	1972.7319	1.717343049	0.014325	up
ENSGALG00000038254	CUZD1	47.6326	551.65385	2.970649573	0.012085	up
ENSGALG00000005753	--	9070.353	4438.6356	-1.007539341	0.049384	down
ENSGALG00000045362	--	114.0793	24.891116	-1.999328998	0.039662	down
ENSGALG00000009572	ADAMTS20	1603.928	4882.5554	1.552622076	0.006469	up
ENSGALG00000002644	RPL10A	33205.31	13719.455	-1.24362494	0.014487	down
ENSGALG00000001288	CFAP74	1603.957	7481.8264	2.014329532	0.042665	up
ENSGALG00000015468	TSTD3	2075.008	613.28062	-1.655949394	0.036826	down
ENSGALG00000038129	PTGES3L	1864.042	860.52265	-1.091376772	0.021825	down
ENSGALG00000005254	--	3992.794	1377.4499	-1.508325196	6.83E-05	down
ENSGALG00000042500	--	44.81443	3.5356459	-3.155934442	0.002099	down
ENSGALG00000001864	C9orf72	2275.335	428.70502	-2.140317653	0.046935	down
ENSGALG00000016722	--	2640.787	866.13719	-1.52410107	0.046197	down
ENSGALG00000002431	--	24505.22	5879.9714	-2.038466649	3.28E-16	down
ENSGALG00000044103	--	68.10159	8.9438728	-2.481201486	0.040106	down
ENSGALG00000026727	SLC30A10	1299.651	3642.7047	1.42561548	0.033194	up
ENSGALG00000015216	SYK	1092.073	220.58751	-2.130027978	0.014515	down
ENSGALG00000006272	--	10911.22	5025.9602	-1.088537069	0.048836	down
ENSGALG00000027665	SYNGR1	35714.81	14374.542	-1.266737115	0.044941	down
ENSGALG00000044365	--	21.70835	145.21912	2.546543288	0.000721	up
ENSGALG00000037438	--	24127.69	7347.0697	-1.680030319	3.60E-05	down
ENSGALG00000042863	SMIM4	2452.694	542.8028	-1.992819717	0.033107	down
ENSGALG00000028770	INHBB	110.3288	13.059888	-2.616149289	0.028048	down
ENSGALG00000013033	--	376.2446	2.9646682	-4.891035255	0.000149	down
ENSGALG00000012200	GCH1	1704.599	360.27349	-2.02653946	0.044599	down
ENSGALG00000044336	--	238.0554	705.31015	1.502256547	0.022462	up
ENSGALG00000035223	NDUFA6	5326.762	1753.5892	-1.539452453	0.016063	down
ENSGALG00000026490	RPS16	67355.27	27675.774	-1.25818717	0.003728	down
ENSGALG00000005958	TRIM66	79181.08	36417.648	-1.103051797	0.004864	down
ENSGALG00000046175	--	277.374	65.691382	-1.992903928	0.000646	down
ENSGALG00000035236	--	8733.185	3733.4828	-1.188372004	0.043825	down
ENSGALG00000016261	CYBB	11289.12	1137.6913	-2.821567969	0.014907	down
ENSGALG00000045860	SOX30	15.82064	57.437456	1.762742345	0.014324	up
ENSGALG00000035485	--	604.753	6.4622386	-3.741554273	0.018092	down
ENSGALG00000041122	SLC17A8	41.09615	2.9203319	-3.040830806	0.018458	down
ENSGALG00000012185	PLA2G12A	10282.24	4968.1175	-1.0253657	0.045226	down
ENSGALG00000030425	--	575.181	121.73687	-2.068441209	0.017448	down
ENSGALG00000010124	RPS8	103787.8	45150.889	-1.182941541	0.001379	down
ENSGALG00000030925	LHPP	23.11215	126.1137	2.205602267	0.029849	up
ENSGALG00000019489	CHST9	293.6943	1111.9554	1.784256505	0.040665	up

ENSGALG00000000812	OAZ1	67806.23	21825.792	-1.546352683	0.046956	down
ENSGALG00000042892	--	3019.06	1413.0247	-1.069486476	0.038588	down
ENSGALG00000040748	--	265.1155	88.107201	-1.511005051	0.041512	down
ENSGALG00000013971	SGK1	11028.73	4849.7192	-1.160002168	0.013833	down
ENSGALG00000039571	PRPH	18894.1	8507.6419	-1.120688149	0.040018	down
ENSGALG00000034177	RPS27L	2227.918	928.37499	-1.220364882	0.047678	down
ENSGALG00000002511	SYCP1	134.9085	328.06632	1.267263358	0.000119	up
ENSGALG00000028302	UQCRQ	5381.642	1012.268	-2.175917244	0.028466	down
ENSGALG00000001096	UQCR11	8747.36	1652.3067	-2.162612677	0.032624	down
ENSGALG00000033736	--	11014.05	667.13214	-3.196570388	0.015897	down
ENSGALG00000030933	C1H12ORF73	1062.295	323.81351	-1.624680525	0.028466	down
ENSGALG00000043793	NLGN2	162.3093	339.3349	1.03750277	0.039241	up
ENSGALG00000014554	ATN1	1247.48	2832.5298	1.162163452	0.005794	up
ENSGALG00000041603	--	2.853872	40.172803	3.230550932	0.002951	up
ENSGALG00000012322	KCTD16	721.1016	37.577826	-3.096211127	0.038479	down
ENSGALG00000006179	RPL13	133582	58450.696	-1.183628131	4.39E-07	down
ENSGALG00000024024	TGFA	233.1965	556.92869	1.223329809	0.014303	up
ENSGALG00000006064	TEKT1	2003.368	4793.8663	1.230111503	0.010402	up
ENSGALG00000033314	--	13.07806	0.6576821	-2.987359117	0.046197	down
ENSGALG00000002988	PHGDH	8705.414	213.91944	-3.825433566	0.006707	down
ENSGALG00000044733	--	448.3041	55.562023	-2.624677581	0.016664	down
ENSGALG00000010868	MACC1	18.89906	302.07822	3.253161462	0.008511	up
ENSGALG00000001655	SPCS1	10633.5	4985.2911	-1.075722485	0.006616	down
ENSGALG00000041266	CNN1	681.0383	12151.158	3.271986987	0.013633	up
ENSGALG00000030372	MPZ	4.65196	246.04354	4.879051668	4.39E-07	up
ENSGALG00000046191	--	0.276083	10.614361	3.469763241	0.016849	up
ENSGALG00000013207	CTNNAL1	12824.67	32554.318	1.299005054	0.03185	up
ENSGALG00000010891	ABCB11	2083.726	190.68147	-2.894976569	0.015916	down
ENSGALG00000008684	EIF4A2	387574.8	176195.65	-1.115703992	0.012087	down
ENSGALG00000041978	K123	226.4143	35.133105	-2.319779707	0.049584	down
ENSGALG00000032371	--	79.59285	12.359031	-2.482264033	0.001951	down
ENSGALG00000014455	LPAR5	135.8695	19.637515	-2.491443108	0.012763	down
ENSGALG00000002578	--	616.4135	251.79552	-1.250976441	0.032488	down
ENSGALG00000012106	SCTR	359.7015	2266.2495	2.315087661	0.041989	up
ENSGALG00000003348	--	2358.361	515.63212	-2.09193944	0.001007	down
ENSGALG00000014955	--	6336.451	14248.138	1.144969336	0.013363	up
ENSGALG00000001634	RPL23	52978.5	22189.088	-1.231464523	0.00443	down
ENSGALG00000045430	--	1939.722	6222.1011	1.620172315	0.006707	up
ENSGALG00000014953	RWDD1	6336.451	14248.138	1.144969336	0.013363	up
ENSGALG00000015461	--	3070.954	189.43437	-3.201096601	0.014303	down
ENSGALG00000009879	--	7006.405	3122.5349	-1.153412805	0.000134	down
ENSGALG00000033001	ATP5J2	14855.93	3514.0834	-1.899497285	0.049384	down
ENSGALG00000035235	--	254.0283	17.568652	-3.148182351	0.011645	down
ENSGALG00000034478	CCL4	153.0763	21.302904	-2.52923422	0.012974	down
ENSGALG00000000399	DUSP28	203.8302	24.231465	-2.684210029	0.013221	down
ENSGALG00000008836	ATP6AP1	20203.95	8224.9779	-1.275046791	0.001007	down
ENSGALG00000011696	JAG2	8025.203	20878.398	1.33163186	0.030386	up
ENSGALG00000010736	DIO1	119.8955	24.825595	-2.097005422	0.01692	down
ENSGALG00000031637	ICOSLG	1760.789	482.08924	-1.770276339	0.015607	down
ENSGALG00000015082	RPS6	80614.93	30775.467	-1.363299554	0.000879	down
ENSGALG00000040915	--	1629.281	83.393059	-3.530324639	0.002728	down
ENSGALG00000001583	TRAF1	1281.396	419.00246	-1.52624857	0.047398	down
ENSGALG00000008753	FBXO48	2459.498	643.80903	-1.825359284	0.015436	down
ENSGALG00000007226	OSTN	1833.719	11447.949	2.467284028	0.001076	up

ENSGALG00000031270	CSK	5196.911	2372.8444	-1.108820159	0.014435	down
ENSGALG00000031130	GGH	2598.672	720.75766	-1.793562992	0.000451	down
ENSGALG00000002932	NME2	55369.75	27023.466	-1.022856221	0.001962	down
ENSGALG00000005997	WDR44	9458.159	25383.537	1.377993189	0.018051	up
ENSGALG00000030044	--	39.92569	1355.9274	4.652509807	8.47E-11	up
ENSGALG00000009601	NELL2	51286.45	7595.5363	-2.495798956	0.007397	down
ENSGALG00000031693	KCNB2	2331.552	814.30258	-1.470141581	0.008511	down
ENSGALG00000009239	--	1788.64	471.8331	-1.788482102	0.038588	down
ENSGALG00000010820	CHGA	33990.02	3300.8638	-2.698961337	0.047042	down
ENSGALG00000006297	TRPC7	467.683	2419.1571	2.197682646	0.00826	up
ENSGALG00000000242	EBF2	384.0825	1396.7472	1.753598922	0.024805	up
ENSGALG00000017392	SPDYA	204.4809	539.9692	1.348517046	0.03218	up
ENSGALG00000038636	LAPTM5	16788.96	3023.9548	-2.257773356	0.014303	down
ENSGALG00000030666	--	19781.99	7898.095	-1.306597982	0.000111	down
ENSGALG00000029509	NECAB1	5875.456	402.23149	-3.039505033	0.025319	down
ENSGALG00000026426	OTUD1	6789.717	3165.8542	-1.078907806	0.018996	down
ENSGALG00000038855	--	5681.114	15176.681	1.366720768	0.028466	up
ENSGALG00000009020	JAG1	8014.845	19408.052	1.258525072	0.000238	up
ENSGALG00000036659	NDUFAF8	1402.754	521.7794	-1.371788105	0.035221	down
ENSGALG00000007986	MOCS3	428.9039	183.92675	-1.182791395	0.042731	down
ENSGALG00000032524	--	62.0884	6.9708265	-2.726267841	0.013741	down
ENSGALG00000017644	COTL1	19397.03	5593.5649	-1.722909157	0.005308	down
ENSGALG00000006771	RPS15A	67719.08	28732.938	-1.221766917	0.000143	down
ENSGALG00000015143	TTR	1621.24	158.78563	-2.759243533	0.030941	down
ENSGALG00000031038	RPL28	51995.54	21865.811	-1.217457574	0.019937	down
ENSGALG00000016721	PLCXD1	2640.787	866.13719	-1.52410107	0.046197	down
ENSGALG00000020402	EFCAB11	508.6553	1061.6649	1.038312466	0.034946	up
ENSGALG00000039114	--	2304.996	1004.6526	-1.173997365	0.009097	down
ENSGALG00000031597	HBM	856.2803	112.13997	-2.732670464	0.000243	down
ENSGALG00000005972	KBTBD12	584.3975	2498.8718	1.953446963	0.018116	up
ENSGALG00000026761	LIN28B	919.1486	3372.8192	1.786753609	0.008683	up
ENSGALG00000017183	MMP13	57.49386	410.16568	2.52926445	0.011573	up
ENSGALG00000008660	CST7	219.2746	26.883378	-2.592391929	0.026832	down
ENSGALG00000046262	CCDC60	238.0784	1078.0471	2.107199027	3.41E-05	up
ENSGALG00000042847	--	651.6236	130.46544	-2.182371614	0.003178	down
ENSGALG00000030900	NCMAP	2801.116	1179.4868	-1.210951876	0.035125	down
ENSGALG00000031099	COX6B1	11415.63	2631.1806	-1.935720578	0.04309	down
ENSGALG00000038405	CASR	345.5324	65.508427	-2.151713053	0.0369	down
ENSGALG00000014754	--	3382.229	379.01353	-2.635903578	0.035221	down
ENSGALG00000005499	IL17RD	2241.358	6052.9378	1.389026155	0.014104	up
ENSGALG00000027932	--	45.48382	370.33194	2.802929211	0.000218	up
ENSGALG00000038387	--	93244.47	19440.079	-2.07130408	0.02546	down
ENSGALG00000036630	--	19.51682	303.91234	3.003607259	0.03611	up
ENSGALG00000040829	--	450.7145	1866.8898	1.884045835	0.04309	up
ENSGALG00000016511	ADGRG2	481.0379	2458.4137	2.253169032	0.000134	up
ENSGALG00000023531	--	549.6705	1153.9484	1.048952872	0.020261	up
ENSGALG00000000558	SLC1A6	2208.94	341.36879	-2.592809366	5.22E-07	down
ENSGALG00000033226	FAM107B	8725.185	3087.4499	-1.437165621	0.031519	down
ENSGALG00000041294	COQ2	966.7869	466.58973	-1.03561181	0.006707	down
ENSGALG00000012061	--	5369.084	643.06352	-2.865048524	4.49E-05	down
ENSGALG00000004116	TRPM8	318.3648	853.99977	1.383838642	0.00763	up
ENSGALG00000029151	ISLR2	400.9854	26.430563	-3.096474423	0.02029	down
ENSGALG00000027973	CATHL1	32.86778	1.9126247	-2.992230957	0.045034	down
ENSGALG00000013193	IRX2	390.8143	45.777235	-2.755232735	0.005321	down

ENSGALG00000019060	MMP27	412.1843	30.587334	-3.137901083	0.007537	down
ENSGALG00000035996	COX6C	22789.97	5465.1087	-1.907482041	0.030386	down
ENSGALG00000011141	ITGB6	2109.036	7001.121	1.656735896	0.012133	up
ENSGALG00000003602	--	1449.226	3073.8103	1.067332912	0.008377	up
ENSGALG00000006358	TMEM86A	3095.812	926.38364	-1.685956656	0.001679	down
ENSGALG00000031181	--	190.1208	93.93178	-1.001038226	0.02738	down
ENSGALG00000005258	SSTR5	942.9773	3014.6017	1.596556829	0.024372	up
ENSGALG00000028706	F-KER	0	10.609262	3.626604917	0.024372	up
ENSGALG00000012770	MAK	3155.801	10171.431	1.622415571	0.009311	up
ENSGALG00000009652	--	279.6232	1720.393	2.33070204	0.025829	up
ENSGALG00000026978	RPL29	50452.73	16198.891	-1.595515724	0.001084	down
ENSGALG00000005918	--	6086.216	18114.747	1.513096103	0.016415	up
ENSGALG00000027611	MRPL54	4384.582	1822.2708	-1.234537536	0.016864	down
ENSGALG00000002679	GLT1D1	2586.989	461.45805	-2.190707062	0.048664	down
ENSGALG00000009091	TRUB1	10779.45	23886.569	1.120067608	0.030386	up
ENSGALG00000030878	RPLP1	80346.74	37735.603	-1.072213699	0.00911	down
ENSGALG00000012173	LGALS3	1930.542	359.02036	-2.180695124	0.031815	down
ENSGALG00000040995	--	413.5486	1977.2596	2.092819183	0.013598	up
ENSGALG00000007699	RPS25	72855.28	34926.573	-1.04776444	0.00197	down
ENSGALG00000039264	SLA	1979.827	411.42071	-2.043491722	0.0452	down
ENSGALG00000041453	--	7123.514	2715.8285	-1.358442284	0.00443	down
ENSGALG00000017330	RPS3	85519.1	36282.879	-1.215438791	0.002803	down
ENSGALG00000010137	TTC6	1808.904	8336.5014	2.015146968	0.032624	up
ENSGALG00000035016	ID2	6326.411	2686.4771	-1.21614361	0.001333	down
ENSGALG00000016555	HADHB	24807.15	10148.585	-1.252569914	0.023921	down
ENSGALG00000011450	--	432.0417	205.52161	-1.046537033	0.047394	down
ENSGALG00000005922	RPL5	445310.7	197091.12	-1.164380341	3.67E-05	down
ENSGALG00000026300	--	3968.689	1078.4309	-1.783683925	0.012974	down
ENSGALG00000030086	ATP5MC2	5222.846	1526.0182	-1.663018987	0.046033	down
ENSGALG00000024398	RPS28	27573.84	11407.141	-1.237945004	0.023649	down
ENSGALG00000005948	RPL27A	79181.08	36417.648	-1.103051797	0.004864	down
ENSGALG00000008417	CFAP58	740.4329	4079.0009	2.327346906	0.000631	up
ENSGALG00000043420	--	0.276083	11.104089	3.418689397	0.022462	up
ENSGALG00000011395	SARAF	8089.354	2711.3024	-1.515603046	0.016849	down
ENSGALG00000043425	--	653.1772	1676.4364	1.328435608	0.004767	up
ENSGALG00000001124	LHX2	197.8913	7.4044255	-3.705267431	0.003612	down
ENSGALG00000040518	--	6.908151	61.61138	2.633009067	0.03558	up
ENSGALG00000010032	--	350.6136	1871.9029	2.227552539	0.010048	up
ENSGALG00000034899	NKAIN3	789.5682	41.902195	-3.543488522	0.001679	down
ENSGALG00000016476	TTC32	6720.177	3201.4365	-1.056400558	0.001951	down
ENSGALG00000015028	RLN3	1645.798	147.37942	-2.745172709	0.048836	down
ENSGALG00000004518	--	1037.466	82.729177	-2.881662911	0.035221	down
ENSGALG00000010342	OTOGL	325.9361	1142.2059	1.743884652	0.002803	up
ENSGALG00000032957	NOCT	3626.353	9024.8183	1.270256331	0.040106	up
ENSGALG00000039991	--	56.77592	196.46026	1.719008302	0.006033	up
ENSGALG00000015594	TRMT44	2591.112	8232.9764	1.596141822	0.01636	up
ENSGALG00000010077	RPS3A	117208.1	52608.412	-1.137839368	0.003158	down
ENSGALG00000031712	--	9436	1958.8476	-2.050056038	0.041448	down
ENSGALG00000027887	--	496.0204	3816.5102	2.533770537	0.029	up
ENSGALG00000031293	SLCO1A2	2616.835	301.12693	-2.630982169	0.030941	down
ENSGALG00000008818	IDH1	25988.67	6563.4555	-1.835691832	0.041512	down
ENSGALG00000013821	GEMIN6	524.5279	204.13177	-1.325208168	0.01284	down
ENSGALG00000004588	RPS14	80680.52	27293.596	-1.545556465	8.91E-08	down
ENSGALG00000001168	POLR2C	8094.049	3475.9564	-1.186752621	0.028222	down

ENSGALG00000033633	--	1732.388	482.66346	-1.72100565	0.043133	down
ENSGALG00000026470	BOD1L1	9276.276	22671.567	1.253720438	0.020313	up
ENSGALG00000035662	--	68.13595	4.4629096	-3.589208868	2.85E-06	down
ENSGALG00000036990	DECR1	14201.16	6206.092	-1.171075732	0.007984	down
ENSGALG00000006804	ODF2L	787.9737	3116.4959	1.907459581	0.001047	up
ENSGALG00000016285	--	6208.052	2846.6641	-1.105718642	0.007101	down
ENSGALG00000037322	HIST1H46	24968.72	9346.3501	-1.36955581	0.022589	down
ENSGALG00000039086	--	0	51.201363	5.010227387	0.000252	up
ENSGALG00000011633	SLC5A8	4.23228	278.33546	4.550849586	0.000243	up
ENSGALG00000046435	--	12.91621	89.09354	2.486929861	0.013538	up
ENSGALG00000015812	SMIM8	468.8552	151.67672	-1.575330899	0.004564	down
ENSGALG00000016919	ACOD1	1588.35	228.20644	-2.389432186	0.048533	down
ENSGALG00000043955	--	10.03503	126.48537	3.231206135	0.000585	up
ENSGALG00000008554	IL17REL	1032.513	118.46212	-2.609173622	0.037142	down
ENSGALG00000034294	ATP6V0D2	401.7044	27.35076	-2.955501537	0.039194	down
ENSGALG00000034280	--	2503.554	1143.3755	-1.11766002	0.000358	down
ENSGALG00000005601	--	1571.926	6719.7369	2.011553728	0.000646	up
ENSGALG00000033066	--	2.07618	17.936374	2.683424944	0.015839	up
ENSGALG00000007418	CD3D	2023.306	225.60611	-2.649070946	0.032624	down
ENSGALG00000013281	--	389.3108	1249.0798	1.598822459	0.027371	up
ENSGALG00000042487	--	16072.45	6711.9337	-1.218157217	0.046956	down
ENSGALG00000032558	INSC	778.0276	2078.4184	1.372971144	0.015949	up
ENSGALG00000023554	PIGW	370.7725	128.83285	-1.494671221	0.000448	down
ENSGALG00000040117	--	284.0461	13.796045	-3.089183512	0.043864	down
ENSGALG00000042362	--	685.8877	4397.9422	2.404545577	0.015839	up
ENSGALG00000003604	SLC46A1	440.4102	205.07011	-1.080792823	0.021286	down
ENSGALG00000004343	HPD	1635.611	62.793413	-3.685810329	0.003979	down
ENSGALG00000005541	GJB1	884.0918	128.2288	-2.474267838	0.016497	down
ENSGALG00000004705	BUD31	6362.739	2851.2183	-1.130747825	0.025576	down
ENSGALG00000025999	NDUFA4	42373.22	8084.2775	-2.12802327	0.046935	down
ENSGALG00000000944	SUPT4H1	3476.145	1618.503	-1.08286247	0.01227	down
ENSGALG00000036396	DPM3	728.4784	276.50359	-1.374177883	0.000335	down
ENSGALG00000004952	--	39376.87	14431.526	-1.435364886	7.41E-09	down
ENSGALG00000014435	MRPL51	2609.133	1184.3784	-1.11498544	0.019159	down
ENSGALG00000016979	SLC25A30	1525.256	3795.5766	1.293610303	0.000646	up
ENSGALG00000037017	C14orf2	7043.207	1584.4319	-1.972326734	0.035125	down
ENSGALG00000014833	RPL37	59485.09	21794.698	-1.417822831	0.001246	down
ENSGALG00000041562	--	31639.67	14801.988	-1.071393367	0.031519	down
ENSGALG00000016986	LCP1	17981.7	2823.3761	-2.448300377	0.005145	down
ENSGALG00000006246	--	10911.22	5025.9602	-1.088537069	0.048836	down
ENSGALG00000041230	--	23594.62	7230.3982	-1.651426057	0.002596	down
ENSGALG00000040363	ABHD5	3523.348	1384.8028	-1.304807602	0.024372	down
ENSGALG00000004917	--	841.5316	40.414584	-3.172581672	0.032688	down
ENSGALG00000017089	SLC46A3	2343.585	443.08567	-2.198648029	0.016243	down
ENSGALG00000009260	TMEM62	5690.898	18639.368	1.630510512	0.019937	up
ENSGALG00000011682	CNOT4	3159.257	10325.123	1.642988242	0.007314	up
ENSGALG00000026578	--	17720.61	63261.169	1.776592109	0.000892	up
ENSGALG00000043828	HEXA	5622.15	2495.469	-1.152396425	0.004098	down
ENSGALG00000002695	SLC15A4	5509.475	2448.5387	-1.161463426	4.63E-07	down
ENSGALG00000015677	SUSD1	1030.029	398.26067	-1.33226982	0.014493	down
ENSGALG00000030430	--	56.32398	0	-5.961947529	1.07E-07	down
ENSGALG00000008587	NDUFAF1	6339.722	2736.7474	-1.179225484	0.03036	down
ENSGALG00000003221	WDR61	4351.678	1994.4927	-1.102836719	0.016337	down
ENSGALG00000043250	--	1583.707	7088.4912	2.07516973	0.000373	up

ENSGALG00000044585	--	105.9529	378.75695	1.761993252	0.003512	up
ENSGALG00000040522	EIF3H	31995.55	14779.507	-1.102046276	0.000368	down
ENSGALG00000041073	--	22.13124	1.0277291	-3.270919492	0.016849	down
ENSGALG00000029856	TOMM7	32677.58	8185.266	-1.839135309	0.046956	down
ENSGALG00000012126	CFI	10282.24	4968.1175	-1.0253657	0.045226	down
ENSGALG00000042366	--	4431.136	1820.8546	-1.263456037	0.000567	down
ENSGALG00000031694	--	2901.231	484.16555	-2.399506396	0.002655	down
ENSGALG00000031792	--	13.17105	0.370047	-3.469606412	0.016323	down
ENSGALG00000035010	--	18.38727	57.679871	1.541726036	0.047867	up
ENSGALG00000012392	IYD	49.96772	1.6673576	-3.32081546	0.030779	down
ENSGALG00000006453	TF	49011.91	3538.0788	-3.492154143	2.85E-06	down
ENSGALG00000036935	CCDC86	1606.301	745.19231	-1.086685556	0.015916	down
ENSGALG00000036765	--	18894.1	8507.6419	-1.120688149	0.040018	down
ENSGALG00000035614	--	10476.83	953.5199	-2.857160897	0.022462	down
ENSGALG00000001983	FNDC7	378.6683	954.47678	1.284210304	0.044038	up
ENSGALG00000002150	CPEB1	4508.661	19373.038	1.961702143	0.01692	up
ENSGALG00000031101	--	123.561	21.887566	-2.275390014	0.01492	down
ENSGALG00000008618	UPF3B	36676.73	17831.588	-1.02885657	0.001275	down
ENSGALG00000037748	--	5542.188	2348.5176	-1.200016609	0.043864	down
ENSGALG00000011472	RPL37A	106260	31296.184	-1.734986971	4.46E-07	down
ENSGALG00000035998	--	30598.94	6126.9429	-2.090878125	0.039241	down
ENSGALG00000000150	RPL9	109896.9	52897.716	-1.035346209	0.019895	down
ENSGALG00000009135	PLEKHA3	5446.625	12389.539	1.15476511	0.03129	up
ENSGALG00000015537	MAN2B2	8613.408	3391.5835	-1.319645043	0.001333	down
ENSGALG00000027961	PCASP2	4384.582	1822.2708	-1.234537536	0.016864	down
ENSGALG00000038694	--	493.8962	61.941417	-2.585390937	0.023446	down
ENSGALG00000041625	LSM5	2500.342	1198.4664	-1.039593552	0.027371	down

Table S5 KEGG enrichment analysis of DEGs in hypothalamus

Pathway	out	All	Pvalue	Genes
Wnt signaling pathway	4	122	0.001123	ENSGALG00000003015; ENSGALG00000009241; ENSGALG00000011358; ENSGALG00000017242
Riboflavin metabolism	1	6	0.023505	ENSGALG00000037587
ECM-receptor interaction	2	67	0.028056	ENSGALG00000005974; ENSGALG00000009641
Focal adhesion	3	173	0.028915	ENSGALG00000005974; ENSGALG00000009641; ENSGALG00000016558
AGE-RAGE signaling pathway in diabetic complications	2	84	0.042523	ENSGALG00000009641; ENSGALG00000016558
Melanogenesis	2	90	0.048179	ENSGALG00000011358; ENSGALG00000017242
Pantothenate and CoA biosynthesis	1	14	0.054035	ENSGALG00000037587
Nitrogen metabolism	1	16	0.061525	ENSGALG00000037975
Histidine metabolism	1	20	0.076338	ENSGALG00000012377
NOD-like receptor signaling pathway	2	119	0.078982	ENSGALG00000030603; ENSGALG00000038405
Phagosome	2	124	0.084805	ENSGALG00000012119; ENSGALG00000027765
mTOR signaling pathway	2	133	0.095613	ENSGALG00000011358; ENSGALG00000017242
Nicotinate and nicotinamide metabolism	1	29	0.108864	ENSGALG00000037587
Starch and sucrose metabolism	1	30	0.11241	ENSGALG00000037587
Intestinal immune network for IgA production	1	33	0.12297	ENSGALG00000041346
Ferroptosis	1	34	0.126463	ENSGALG00000012298
Arachidonic acid metabolism	1	43	0.157317	ENSGALG00000030886
TGF-beta signaling pathway	1	77	0.264839	ENSGALG00000011274
Pyrimidine metabolism	1	91	0.30523	ENSGALG00000037587
Purine metabolism	1	154	0.462423	ENSGALG00000037587
Regulation of actin cytoskeleton	1	181	0.51893	ENSGALG00000041346
Cytokine-cytokine receptor interaction	1	188	0.532637	ENSGALG00000041346
MAPK signaling pathway	1	249	0.637426	ENSGALG00000016558
Metabolic pathways	2	1110	0.955855	ENSGALG00000030886; ENSGALG00000037587

Table S6 Significantly enriched pathways and five key pathways related to reproduction in ovary

Pathway	out	All	Pvalue	Genes
Ribosome	38	114	1.11E-25	ENSGALG00000000150; ENSGALG00000000474; ENSGALG00000001634; ENSGALG00000002157; ENSGALG00000002644; ENSGALG00000003197; ENSGALG00000003966; ENSGALG00000004588; ENSGALG00000004952; ENSGALG00000005338; ENSGALG00000005922; ENSGALG00000005948; ENSGALG00000006179; ENSGALG00000007611; ENSGALG00000007699; ENSGALG00000008620; ENSGALG00000010077; ENSGALG00000010124; ENSGALG00000011290; ENSGALG00000012229; ENSGALG00000013990; ENSGALG00000014833; ENSGALG00000015082; ENSGALG00000015195; ENSGALG00000015339; ENSGALG00000015617; ENSGALG00000016232; ENSGALG00000016775; ENSGALG00000017330; ENSGALG00000026490; ENSGALG00000026978;
Oxidative phosphorylation	15	115	6.25E-05	ENSGALG00000001096; ENSGALG00000001330; ENSGALG00000007863; ENSGALG00000008836; ENSGALG00000014981; ENSGALG00000015372; ENSGALG00000025999; ENSGALG00000027963; ENSGALG00000028302; ENSGALG00000030086; ENSGALG00000030925; ENSGALG00000033001; ENSGALG00000034294; ENSGALG00000035223; ENSGALG00000035996
Glycine, serine and threonine metabolism	7	36	0.000552248	ENSGALG00000002397; ENSGALG00000002988; ENSGALG00000004491; ENSGALG00000004518; ENSGALG00000008317; ENSGALG00000023435; ENSGALG00000040182

Metabolic pathways	60	1110	0.0102984	ENSGALG00000000549;<br>ENSGALG00000001096;<br>ENSGALG00000001168;<br>ENSGALG00000001330;<br>ENSGALG00000002397;<br>ENSGALG00000002932;<br>ENSGALG00000002988;<br>ENSGALG00000004343;<br>ENSGALG00000004438;<br>ENSGALG00000004491;<br>ENSGALG00000004518;<br>ENSGALG00000004875;<br>ENSGALG00000004947;<br>ENSGALG00000006300;<br>ENSGALG00000006841;<br>ENSGALG00000007863;<br>ENSGALG00000008317;<br>ENSGALG00000008818;<br>ENSGALG00000008836;<br>ENSGALG00000009511;<br>ENSGALG00000010073;<br>ENSGALG00000011093;<br>ENSGALG00000011203;<br>ENSGALG00000011450;<br>ENSGALG00000012200;<br>ENSGALG00000012291;<br>ENSGALG00000012550;<br>ENSGALG00000012748;<br>ENSGALG00000013094;<br>ENSGALG00000013135;<br>ENSGALG00000013828;
Lysosome	10	108	0.01304888	ENSGALG00000004438;<br>ENSGALG00000004769;<br>ENSGALG00000006378;<br>ENSGALG00000008836;<br>ENSGALG00000010811;<br>ENSGALG00000026364;<br>ENSGALG00000030016;<br>ENSGALG00000034294;<br>ENSGALG00000038636;<br>ENSGALG00000043828
RNA polymerase	4	23	0.01346846	ENSGALG00000001168;<br>ENSGALG00000004947;<br>ENSGALG00000006841;<br>ENSGALG00000012291
Cardiac muscle contraction	6	56	0.02676573	ENSGALG00000000302;<br>ENSGALG00000001096;<br>ENSGALG00000007863;<br>ENSGALG00000027963;<br>ENSGALG00000028302;<br>ENSGALG00000035996
Glycosaminoglycan degradation	3	17	0.03074922	ENSGALG00000004438;<br>ENSGALG00000011203;<br>ENSGALG00000043828

Metabolism of xenobiotics by cytochrome P450	4	32	0.0410561	ENSGALG00000003445; ENSGALG00000009803; ENSGALG00000013098; ENSGALG00000028858
Ferroptosis	4	34	0.04970595	ENSGALG00000006453; ENSGALG00000012550; ENSGALG00000014570; ENSGALG00000016261
Folate biosynthesis	2	26	0.2919325	ENSGALG00000012200; ENSGALG00000031130
Steroid hormone biosynthesis	2	33	0.3983711	ENSGALG00000013063; ENSGALG00000028858
Steroid biosynthesis	1	17	0.5124244	ENSGALG00000006378
Oocyte meiosis	3	93	0.7467628	ENSGALG00000002150; ENSGALG00000014228; ENSGALG00000017392
Progesterone-mediated oocyte maturation	2	77	0.83463	ENSGALG00000002150; ENSGALG00000017392
